# Supplementary material for: Screening and Activity Analysis of α-Glucosidase Inhibitory Peptides Derived from Coix Seed Prolamins Using Bioinformatics and Molecular Docking
Source: Foods. 2023 Oct 30;12(21):3970. doi: 10.3390/foods12213970 (PMC10649794; doi:10.3390/foods12213970)
Supplement: Supplementary file 1 [file foods-12-03970-s001.zip › Figure S1 and Table S1.pdf]

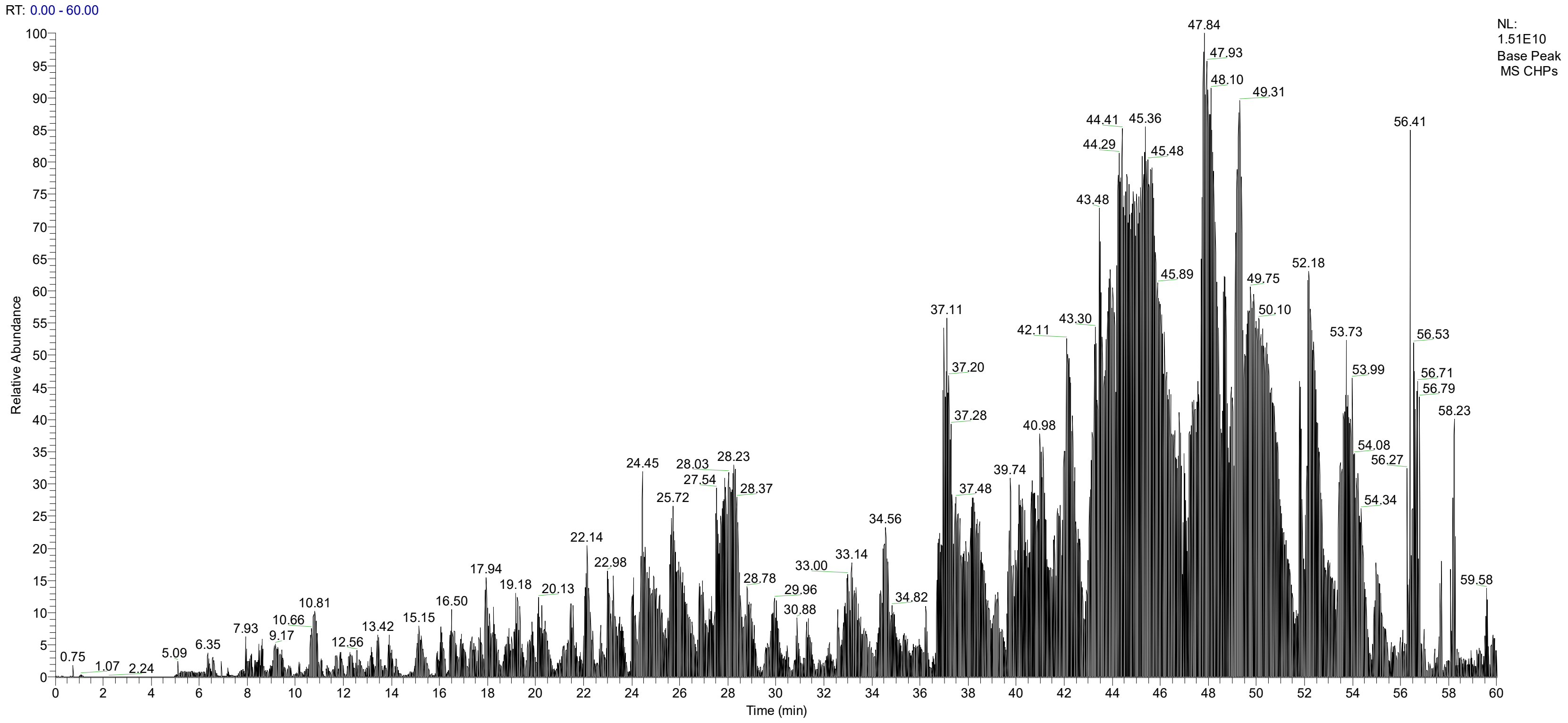

Figure S1. Basepeak diagram of CHPs mass spectrum

Table S1. Peptides identified from CHPs and their bioinformatics analysis

No.	Sequence	Length	Molecular weight/Da	Peptide Ranker	Iso-electric point (pI)	Net Charge at pH7.0	Hydrophobicity /(kcal·mol^{-1})	Toxicy	Allergenicity	Protein	Affinity(kcal/mol)
1	FLSPF	5	609.31625	0.964433	5.41	0	3.83	-	+	α-coixin	-8.5
2	FPCNPL	6	746.34215	0.953249	5.2	0	6.05	-	+	α-coixin	-7.6
3	QQFPFN	6	779.36024	0.906747	5.33	0	7.01	-	+	α-coixin	-7.9
4	CSQRPLLPPFLP	12	1423.7646	0.904167	9.5	1	6.02	-	+	α-coixin	-8
5	FPSNPL	6	673.34353	0.898827	5.52	0	6.53	-	-	α-coixin	-7.2
6	GCQPPHLPPPPV	12	1294.6492	0.89616	7.17	0	11.26	-	-	γ-coixin	-8.3
7	LFPCNPL	7	859.42621	0.887747	5.21	0	4.8	-	-	β-coixin	-7.1
8	AAIIPRFL	8	899.55927	0.886889	11.18	1	5.65	-	-	β-coixin	-7.5
9	QQQQLFPF	8	1034.5185	0.875036	5.38	0	6.45	-	+	α-coixin	-7.6
10	QSQLFPC	7	878.39564	0.873712	4.93	0	7.06	-	-	α-coixin	-7.9
11	SQLFPCNPL	9	1074.5168	0.86338	5.2	0	6.03	-	+	α-coixin	-7.6
12	QQQQFLPF	8	1034.5185	0.861822	5.38	0	6.45	-	+	α-coixin	-7.6
13	FPCNPLV	7	845.41056	0.852117	5.24	0	5.59	-	-	α-coixin	-8.8
14	LPPFLPS	7	769.43743	0.850373	5.45	0	4.57	-	-	α-coixin	-8.4
15	QQQFPFN	7	907.41882	0.847167	5.33	0	7.78	-	+	α-coixin	-8.2
16	QFLPAL	6	687.39556	0.842742	5.48	0	5.1	-	-	α-coixin	-7.8
17	QQLLPF	6	744.41703	0.829531	5.38	0	5.37	-	+	α-coixin	-7.5
18	CSLAPTAAIIPRFL	14	1528.8436	0.826699	9.5	1	5.73	-	-	α-coixin	-6.3
19	LLPFYPN	7	862.45889	0.81539	5.39	0	4.11	-	+	α-coixin	-8.7
20	QQQLLLPF	8	985.55967	0.81471	5.38	0	4.89	-	+	α-coixin	-7.7
21	QRPLLPPFLPS	11	1263.7339	0.805662	10.8	1	6.04	-	-	α-coixin	-7.7

22	CSQRPLLPPFLPSVT	15	1710.9127	0.803383	9.5	1	6.27	-	-	α-coixin	-7.5
23	LFPSNPL	7	786.42759	0.801491	5.58	0	5.28	-	-	α-coixin	-8
24	SQQQQFLPF	9	1121.5506	0.792348	5.39	0	6.91	-	+	α-coixin	-8.2
25	QRQLLPF	7	900.51814	0.78954	10.8	1	7.18	-	+	α-coixin	-7.9
26	CSQRPLLPPFLPS	13	1510.7966	0.776267	9.5	1	6.48	-	+	α-coixin	-7.4
27	SWQQPIVGRVF	11	1315.7037	0.77438	10.85	1	7.16	-	-	α-coixin	-6.9
28	SQQFLPAL	8	902.48617	0.770132	5.5	0	6.33	-	+	α-coixin	-7.4
29	SWQQPIVGGVGF	12	1273.6455	0.768389	5.39	0	7.65	-	-	α-coixin	-7.4
30	WQQPIVGGVGF	11	1186.6135	0.767878	5.42	0	7.19	-	-	α-coixin	-8.1
31	CQQPIVGAALF	11	1202.6118	0.762157	5.13	0	7.17	-	+	α-coixin	-7.8
32	QPHPCPYQPQHPSPF	15	1815.8151	0.753274	7.3	0	13.73	-	+	γ-coixin	-8
33	QLLPFYPN	8	990.51747	0.752167	5.32	0	4.88	-	+	α-coixin	-8.3
34	QQFLPAL	7	815.45414	0.749964	5.48	0	5.87	-	-	α-coixin	-7.5
35	SQRPLLPPFLPS	12	1350.766	0.748206	10.85	1	6.5	-	-	α-coixin	-7.4
36	FPCNPLVA	8	916.44767	0.745656	5.21	0	6.09	-	-	α-coixin	-8.4
37	LSWQQPIVGGVGF	13	1386.7296	0.743765	5.46	0	6.4	-	+	α-coixin	-7.9
38	RPLLPPFLPS	10	1135.6754	0.740627	10.73	1	5.27	-	-	α-coixin	-8.1
39	LLPFYPQ	7	876.47454	0.736715	5.42	0	4.03	-	-	α-coixin	-7.4
40	IIPRFLPH	8	991.59672	0.730784	11.12	1	7.12	-	+	α-coixin	-8.1
41	QQRQLLPF	8	1028.5767	0.730333	10.8	1	7.95	-	+	α-coixin	-7.2
42	QQQQFLPAL	9	1071.5713	0.718178	5.48	0	7.41	-	+	α-coixin	-7.6
43	QLFPSNPL	8	914.48617	0.714022	5.48	0	6.05	-	-	α-coixin	-8.3
44	QQLLPFYPN	9	1118.576	0.712016	5.32	0	5.65	-	+	α-coixin	-7
45	QQQLLPFYPN	10	1246.6346	0.711837	5.32	0	6.42	-	-	α-coixin	-7.3
46	QRQLLPFN	8	1014.5611	0.711825	10.8	1	8.03	-	+	α-coixin	-7.5
47	RFLPPV	6	727.4381	0.711307	10.73	1	6.57	-	+	α-coixin	-8.1
48	SQLFPCNPLVA	11	1244.6223	0.710979	5.21	0	6.07	-	-	α-coixin	-7.6
49	QQQFLPSL	8	959.50763	0.70839	5.48	0	6.6	-	+	α-coixin	-7.3
50	LSWQQPIVGRVF	12	1428.7878	0.707602	11.11	1	5.91	-	-	α-coixin	-7.5

51	SWQQPIVGR	9	1069.5669	0.703379	10.85	1	9.33	-	-	α-coixin	-8.4
52	QQQQLLPFYPN	11	1374.6932	0.699724	5.32	0	7.19	-	+	α-coixin	-7.6
53	SQLFPSNPL	9	1001.5182	0.696191	5.5	0	6.51	-	+	α-coixin	-7.1
54	ATPVFLL	7	759.45308	0.692888	5.59	0	4.12	-	+	γ-coixin	-8
55	QSQLFPSNPL	10	1129.5768	0.692315	5.48	0	7.28	-	+	α-coixin	-7.2
56	FSSNPLA	7	734.3599	0.691488	5.53	0	7.35	-	+	α-coixin	-7.7
57	AVARLFL	7	788.49086	0.685505	11.18	1	6.04	-	-	α-coixin	-7.5
58	SQLFPCNPLVAA	12	1315.6595	0.683097	5.21	0	6.57	-	+	α-coixin	-7
59	RFLPPVSAIGF	11	1202.6812	0.678841	10.73	1	5.85	-	-	α-coixin	-8.3
60	AQLFPSNPL	9	985.52328	0.677206	5.59	0	6.55	-	+	α-coixin	-7.7
61	VIIPQCSLAPTAAIIPR	17	1819.039	0.674531	9.02	1	6.9	-	-	α-coixin	-6.9
62	QQQQFLPSL	9	1087.5662	0.674033	5.48	0	7.37	-	+	α-coixin	-6.7
63	LQQFLPAL	8	928.5382	0.67313	5.58	0	4.62	-	+	α-coixin	-7.5
64	QLFPCNPLVA	10	1157.5903	0.671625	5.2	0	5.61	-	+	α-coixin	-7.6
65	RNPAASCQQPIVGAALF	17	1798.9148	0.669429	8.65	1	11.43	-	-	α-coixin	-7.3
66	WQQPIVGR	8	982.53485	0.66463	10.95	1	8.87	-	-	α-coixin	-8.1
67	QLPINPL	7	793.46979	0.663923	5.48	0	6.18	-	-	α-coixin	-7.4
68	WQQPIVGG	8	883.4552	0.66356	5.55	0	8.21	-	-	α-coixin	-8.3
69	QLLPFYPQ	8	1004.5331	0.66325	5.34	0	4.8	-	+	α-coixin	-8
70	QQRQLLPFN	9	1142.6196	0.662547	10.8	1	8.8	-	+	α-coixin	-
71	LFPCNPLVA	9	1029.5317	0.662424	5.22	0	4.84	-	+	α-coixin	-8.1
72	LSWQQPIVGG	10	1083.5713	0.657849	5.6	0	7.42	-	+	α-coixin	-8.3
73	AAIIPRFLAPV	11	1166.7176	0.654929	11.18	1	5.83	-	+	β-coixin	-8.3
74	SQQFLPA	7	789.4021	0.65168	5.51	0	7.58	-	+	α-coixin	-7.5
75	TQQLLPF	7	845.4647	0.651234	5.37	0	5.62	-	+	α-coixin	-7.5
76	GVRQPMTSPCPC	12	1388.5999	0.651033	8.28	1	11.59	-	-	γ-coixin	-7.5
77	LFPSNPLA	8	857.4647	0.648003	5.6	0	5.78	-	+	α-coixin	-8.9
78	PALSWQQPIVGGVGF	15	1554.8195	0.644328	5.5	0	7.04	-	-	α-coixin	-7.4
79	QQLLPFYPQ	9	1132.5917	0.642308	5.34	0	5.57	-	+	α-coixin	-7.5

80	ALATPVFL	8	830.49019	0.642242	5.59	0	4.62	-	-	γ-coixin	-7.7
81	YGAGDLM	7	725.30543	0.639497	3.02	-1	11.71	-	+	β-coixin	-7.5
82	HLPFNPQ	7	851.42899	0.639342	7.69	0	9.17	-	+	α-coixin	-8.7
83	SLMNPALSW	9	1017.4954	0.635663	5.6	0	5.05	-	+	α-coixin	-7.3
84	LSWQQPIVGR	10	1182.6509	0.630091	11.11	1	8.08	-	-	α-coixin	-7.7
85	FPSNPLA	7	744.38064	0.630091	5.53	0	7.03	-	-	α-coixin	-7.1
86	WQQPIVG	7	826.43374	0.629698	5.55	0	7.06	-	-	α-coixin	-7.7
87	AIIPQCSLAPSAAIIPQ	17	1748.9495	0.629658	5.11	0	7.03	-	-	α-coixin	-6.7
88	SWQQPIVGGV	10	1069.5556	0.628159	5.54	0	8.21	-	-	α-coixin	-7.7
89	QQLLAFNPL	9	1042.5811	0.627684	5.48	0	5.47	-	+	α-coixin	-7.1
90	CSLAPTAAIIPR	12	1268.6911	0.626589	9.5	1	8.69	-	+	α-coixin	-6.2
91	SCQQPIVGR	9	1043.5182	0.62638	8.76	1	11.4	-	-	α-coixin	-6.9
92	QQLQQFLPAL	10	1184.6554	0.623992	5.48	0	6.16	-	+	α-coixin	-7.5
93	PCNPLVANAAAYL	13	1372.6809	0.622054	5.21	0	8.19	-	-	α-coixin	-7.8
94	QQQLLPFYPQ	10	1260.6503	0.618021	5.34	0	6.34	-	-	α-coixin	-7.6
95	SLMNPAL	7	744.38401	0.616927	5.5	0	6.68	-	-	α-coixin	-7.4
96	QQQRQLLPF	9	1156.6353	0.614182	10.8	1	8.72	-	+	α-coixin	-7.1
97	QQRQLLPFNQM	11	1401.7187	0.61275	10.8	1	8.9	-	-	α-coixin	-4
98	ALRNPAASCQQPIVGAALF	19	1983.036	0.609432	9.09	1	10.68	-	+	α-coixin	-7
99	IIPRFLAPV	9	1024.6433	0.60577	11.12	1	4.83	-	+	β-coixin	-7.7
100	QQLPINPL	8	921.52837	0.603928	5.48	0	6.95	-	+	α-coixin	-7.8
101	AAIIPRFLAPVSAI	14	1437.8708	0.601295	11.18	1	5.6	-	-	β-coixin	-7.4
102	IPRFLPPVSAI	11	1208.7281	0.60059	11.12	1	5.43	-	+	α-coixin	-8.3
103	LFPSNPLVA	9	956.53312	0.600145	5.6	0	5.32	-	-	α-coixin	-7.8
104	QLLPAL	6	653.41121	0.599194	5.48	0	5.56	-	-	α-coixin	-7.8
105	LFPCNPLVAA	10	1100.5689	0.598454	5.22	0	5.34	-	+	α-coixin	-7.4
106	CSCSPVAVPY	10	1138.4787	0.589377	5.02	0	7.93	-	+	β-coixin	-8.3
107	LSWQQPIVGGV	11	1182.6397	0.588817	5.63	0	6.96	-	+	α-coixin	-8.3
108	QQQQLLPFYPQ	11	1388.7089	0.588341	5.34	0	7.11	-	+	α-coixin	-6.8

109	GQCIEFLRHQCSPAATPY	18	2133.9724	0.587626	7.01	0	15.52	-	+	γ-coixin	-6.9
110	QQQQFLPSLSQL	12	1415.7409	0.586533	5.48	0	7.35	-	-	α-coixin	-7
111	QLFPSNPLA	9	985.52328	0.585702	5.49	0	6.55	-	+	α-coixin	-8.4
112	FQQQQQRQLLPF	12	1559.8209	0.584691	10.9	1	8.55	-	-	β-coixin	-6.7
113	IIPQCSQRPLLPPFLPS	17	1962.0761	0.584329	9.03	1	5.15	-	-	α-coixin	-7.1
114	FLQQQQRQLLPF	12	1544.8463	0.581958	10.9	1	6.53	-	+	α-coixin	-6.7
115	MNPALSWQQPIVGGVGF	17	1799.9029	0.581382	5.4	0	7.22	-	+	α-coixin	-7.7
116	LSWQQPIVG	9	1026.5498	0.580399	5.6	0	6.27	-	-	α-coixin	-7.8
117	IIPRFLPPVSAI	12	1321.8122	0.579475	11.12	1	4.31	-	-	α-coixin	-7.5
118	QHLPFNPQ	8	979.48756	0.57884	7.59	0	9.94	-	+	α-coixin	-8
119	SQLFPSNPLVA	11	1171.6237	0.577573	5.51	0	6.55	-	+	α-coixin	-7.5
120	QQPIVGGVGF	10	1000.5342	0.576374	5.38	0	9.28	-	-	α-coixin	-8.2
121	SQLFPSNPLA	10	1072.5553	0.576225	5.51	0	7.01	-	+	α-coixin	#N/A
122	QQHLPFNPQ	9	1107.5461	0.574762	7.59	0	10.71	-	+	α-coixin	-8.4
123	QQPIGQPL	8	879.48142	0.573868	5.48	0	9.27	-	-	γ-coixin	-7.5
124	QLPLVNF	7	829.46979	0.566444	5.38	0	4.99	-	-	α-coixin	-7.7
125	QQQFLPSLSQL	11	1287.6823	0.565906	5.48	0	6.58	-	-	α-coixin	-7.4
126	QQQRQLLPFNQM	12	1529.7773	0.565365	10.8	1	9.67	-	-	α-coixin	-6.9
127	CQQPIVGR	8	956.48618	0.564657	9.5	1	10.94	-	+	α-coixin	-6.5
128	LRNPAASCQQPIVGAALF	18	1911.9989	0.561186	9.02	1	10.18	-	-	α-coixin	-6.6
129	SQLFPSNPLVT	11	1201.6343	0.560072	5.3	0	6.3	-	+	α-coixin	-7.3
130	SQLFPSNPLVAA	12	1242.6608	0.556342	5.51	0	7.05	-	+	α-coixin	-7.2
131	IIPRFLAPVSAI	12	1295.7965	0.554588	11.12	1	4.67	-	-	β-coixin	-7.6
132	QQQLPINPL	9	1049.5869	0.554307	5.48	0	7.72	-	-	α-coixin	-7.4
133	LALRNPAASCQQPIVGAALF	20	2096.1201	0.553395	9.02	1	9.43	-	-	α-coixin	-6.7
134	LQQAIIGGAIF	11	1129.6495	0.55082	5.46	0	6.42	-	+	α-coixin	-7.5
135	AAIIPR	6	639.4068	0.549079	11.18	1	8.61	-	+	α-coixin	-7.1
136	QLFPSNPLVAA	11	1155.6288	0.549028	5.49	0	6.59	-	+	α-coixin	-8.2
137	QQQQLPINPL	10	1177.6455	0.548434	5.48	0	8.49	-	+	α-coixin	-7

138	QLQQLLPAL	9	1022.6124	0.545404	5.48	0	5.85	-	+	α-coixin	-6.3
139	IIPRFLAPVS	10	1111.6754	0.54386	11.12	1	5.29	-	-	β-coixin	-8.1
140	AQLLPFNQQLAVASPIASLQ	20	2108.163	0.542326	5.44	0	6.74	-	+	α-coixin	-7
141	QLQQFLPA	8	943.51272	0.538263	5.49	0	6.64	-	+	α-coixin	-7.3
142	AIIPRFLPPVSAI	13	1392.8493	0.537285	11.18	1	4.81	-	+	α-coixin	-8
143	LFPSNPLVVT	10	1085.6121	0.535907	5.36	0	4.61	-	+	α-coixin	-7
144	QQQQRQLLPFNQM	13	1657.8359	0.534815	10.8	1	10.44	-	-	α-coixin	-6.7
145	LSCQQPIVGR	10	1156.6023	0.534064	9.02	1	10.15	-	-	α-coixin	-6.9
146	AAIIPRFLPPVSAI	14	1463.8864	0.530864	11.18	1	5.31	-	-	α-coixin	-6.9
147	AFLQQQQRQLLPF	13	1615.8835	0.53028	11.18	1	7.03	-	-	α-coixin	-7.1
148	QLFSSNPL	8	904.46543	0.529918	5.48	0	6.37	-	+	α-coixin	-8
149	QQQQRQLLPF	10	1284.6939	0.528584	10.8	1	9.49	-	-	α-coixin	-6.6
150	QQLQQLLPAL	10	1150.671	0.527076	5.48	0	6.62	-	+	α-coixin	-6.8
151	QQAIIGGAIF	10	1016.5655	0.526419	5.38	0	7.67	-	+	α-coixin	-7.2
152	IPRFLPPVSAV	11	1194.7125	0.519018	11.12	1	6.09	-	+	α-coixin	-7.9
153	FPSNPLVTA	9	944.49673	0.512651	5.53	0	6.82	-	+	α-coixin	-7.2
154	RQPQCSCSPVAVPY	14	1647.7497	0.508958	8.18	1	11.42	-	-	β-coixin	-6.9
155	QQQHLPFNPQ	10	1235.6047	0.508282	7.59	0	11.48	-	-	α-coixin	-6.8
156	QLQHILPVL	9	1059.6441	0.507467	7.59	0	6.58	-	+	α-coixin	-7.2
157	QQQQQRQLLPFNQM	14	1785.8944	0.507135	10.8	1	11.21	-	+	β-coixin	-7
158	IIPRFLAPVSAIGFEHPAVQ	20	2161.2048	0.505111	7.9	0	11.02	-	-	β-coixin	-7.2
159	SQLFPSNPLVTA	12	1272.6714	0.50392	5.51	0	6.8	-	+	α-coixin	-7
160	SLMNPALSWQQPIVG	15	1639.8392	0.503075	5.51	0	6.3	-	-	α-coixin	-7.4
161	IIPRFLPPVSA	11	1208.7281	0.502783	11.12	1	5.43	-	+	α-coixin	-7.9
162	SQLFPSNPLVVTN	13	1414.7456	0.500599	5.34	0	6.69	-	+	α-coixin	-8
163	SLMNPALSWQQPIVGGV	17	1795.9291	0.499683	5.54	0	6.99	-	-	α-coixin	-
164	SLMNPALSCQQPIVGR	16	1769.8917	0.498589	8.76	1	10.18	-	-	α-coixin	-
165	IIPRFLPPVSAV	12	1307.7965	0.498178	11.12	1	4.97	-	-	α-coixin	-
166	LFPSNPLVTA	10	1057.5808	0.487906	5.6	0	5.57	-	+	α-coixin	-

167	LQQLLPAL	8	894.55385	0.487367	5.58	0	5.08	-	+	*α*-coixin	-
168	YQQLPSYC	8	1057.4539	0.487151	4.93	0	7.35	-	-	*β*-coixin	-
169	QQQQQRQLLPF	11	1412.7524	0.485675	10.8	1	10.26	-	-	*β*-coixin	-
170	QLQQLLPALSQL	12	1350.7871	0.485359	5.48	0	5.83	-	-	*α*-coixin	-
171	ASCQQPIVGA	10	1029.4913	0.484507	5.22	0	10.59	-	+	*α*-coixin	-
172	QFLPALSQL	9	1015.5702	0.483986	5.48	0	5.08	-	+	*α*-coixin	-
173	TIIPQCSQRPLLPPFLPS	18	2063.1238	0.483834	8.7	1	5.4	-	-	*α*-coixin	-
174	MNPALSCQQPIVGR	14	1569.7756	0.481713	8.79	1	10.97	-	-	*α*-coixin	-
175	ANPITYPA	8	845.42832	0.478355	5.59	0	8.45	-	-	*α*-coixin	-
176	GFEHPAVR	8	911.46135	0.474336	7.91	0	15.29	-	+	*α*-coixin	-
177	RFLPPVS	7	814.47012	0.471512	10.73	1	7.03	-	-	*α*-coixin	-
178	QQFLPALSQL	10	1143.6288	0.470815	5.48	0	5.85	-	+	*α*-coixin	-
179	LQQQQRQLLPF	11	1397.7779	0.468946	11.11	1	8.24	-	+	*α*-coixin	-
180	FLPPVSAIG	9	899.51165	0.468931	5.53	0	5.75	-	+	*α*-coixin	-
181	YGAGDL	6	594.26494	0.4685	3.12	-1	12.38	-	+	*β*-coixin	-
182	QAIIGGAIF	9	888.5069	0.466324	5.38	0	6.9	-	+	*α*-coixin	-
183	AAIIPRFLPPVSAV	14	1449.8708	0.465172	11.18	1	5.97	-	-	*α*-coixin	-
184	LQQQQRQLLPFNQM	14	1770.9199	0.463201	11.11	1	9.19	-	-	*α*-coixin	-
185	QQFLPSLSQL	10	1159.6237	0.463011	5.48	0	5.81	-	+	*α*-coixin	-
186	CSLAPTAAIIPRFLPPVSAV	20	2079.1551	0.45864	9.5	1	6.05	-	-	*α*-coixin	-
187	LSCQQPIVG	9	1000.5012	0.453089	5.22	0	8.34	-	+	*α*-coixin	-
188	QHILPVL	7	818.50142	0.451485	7.59	0	7.06	-	+	*α*-coixin	-
189	QPLDQL	6	712.37555	0.448852	3.12	-1	10.72	-	+	*α*-coixin	-
190	ALRNPATLL	9	967.58147	0.445854	11.18	1	8.2	-	+	*α*-coixin	-
191	LFPSNPLVTAN	11	1171.6237	0.444793	5.41	0	6.42	-	+	*α*-coixin	-
192	TVATLFPL	8	860.50076	0.44477	5.47	0	4.37	-	+	*γ*-coixin	-
193	QQQLLPFNQVAVA	13	1454.7882	0.444727	5.49	0	7.84	-	+	*α*-coixin	-
194	QQPLIQL	7	838.49125	0.432606	5.48	0	6.73	-	+	*α*-coixin	-
195	LQHILPVL	8	931.58549	0.431239	7.89	0	5.81	-	+	*α*-coixin	-

196	QNQELLPTL	9	1054.5659	0.430118	3.2	-1	10.56	-	+	α-coixin	-
197	ANPITYPAA	9	916.46543	0.421128	5.59	0	8.95	-	+	α-coixin	-
198	ILQQPL	6	710.43268	0.410805	5.58	0	5.96	-	+	α-coixin	-
199	IIPQFLPTVSAIGFEHPAVQ	20	2163.1728	0.407779	5.06	-1	9.73	-	+	α-coixin	-
200	QILPVL	6	681.44251	0.403083	5.48	0	4.73	-	+	α-coixin	-
201	QLQQLLPA	8	909.52837	0.398012	5.49	0	7.1	-	+	α-coixin	-
202	IQRFLPPVSAI	11	1239.7339	0.39543	11.12	1	6.06	-	+	α-coixin	-
203	CQQPIVGAA	9	942.4593	0.395195	5.23	0	10.13	-	+	α-coixin	-
204	CQQPIVGA	8	871.42219	0.395114	5.23	0	9.63	-	+	α-coixin	-
205	RFLPPVSAIG	10	1055.6128	0.393757	10.73	1	7.56	-	-	α-coixin	-
206	QQPIVGR	7	796.45554	0.386645	10.8	1	10.96	-	+	α-coixin	-
207	FLPPVSAI	8	842.49019	0.383119	5.51	0	4.6	-	-	α-coixin	-
208	QRFLPPVSAI	10	1126.6499	0.382502	7.8	1	7.18	-	+	α-coixin	-
209	QLQQILPVL	9	1050.6437	0.380952	5.48	0	5.02	-	+	α-coixin	-
210	SILRSSPLFAQQPL	14	1555.8722	0.378886	10.85	1	6.83	-	-	α-coixin	-
211	SLMNPALS	8	831.41604	0.37874	5.38	0	7.14	-	+	α-coixin	-
212	FLPPVSA	7	729.40613	0.377701	5.53	0	5.72	-	-	α-coixin	-
213	QQVLPALSQLVVANPAAY	18	1881.036	0.375659	5.37	0	7.96	-	-	α-coixin	-
214	QQPLDQL	7	840.43413	0.375134	3.12	-1	11.49	-	-	α-coixin	-
215	AVASPIASL	9	827.47527	0.374068	5.59	0	7.63	-	+	α-coixin	-
216	ALRNPATL	8	854.4974	0.372145	11.18	1	9.45	-	-	α-coixin	-
217	TFFPQCSPVTT	11	1283.5856	0.36086	5.04	0	6.26	-	-	α-coixin	-
218	SAAIIPQ	7	698.39629	0.35839	5.37	0	8.03	-	+	α-coixin	-
219	SILQQPLIQL	10	1151.6914	0.352639	5.5	0	4.82	-	-	α-coixin	-
220	ALRNPAASCQQPIVGA	16	1651.8464	0.352506	9.09	1	13.14	-	+	α-coixin	-
221	AVANPNSYLQQQQLLPF	17	1929.9949	0.337652	5.46	0	7.79	-	+	α-coixin	-
222	VIIPQCSL	8	928.50519	0.337595	5.21	0	5.21	-	+	α-coixin	-
223	ALRNPAASCQQPIVGAA	17	1722.8835	0.336699	9.09	1	13.64	-	-	α-coixin	-
224	QQPLAQL	7	796.4443	0.335794	5.48	0	8.35	-	+	α-coixin	-

225	QELLPTL	7	812.46437	0.334795	3.2	-1	8.94	-	-	α-coixin	-
226	LQQILPVL	8	922.58515	0.333711	5.58	0	4.25	-	+	α-coixin	-
227	QLLASNPL	8	854.48617	0.327784	5.48	0	6.87	-	+	α-coixin	-
228	QQLLPALSQL	10	1109.6445	0.326241	5.48	0	6.31	-	+	α-coixin	-
229	VIIPQCSLAPTA	12	1268.6799	0.325034	5.22	0	6.69	-	-	α-coixin	-
230	RFLPPVSAI	9	998.5913	0.323899	10.73	1	6.41	-	+	α-coixin	-
231	VANPITYPA	9	944.49673	0.321849	5.58	0	7.99	-	-	α-coixin	-
232	ALRNPA	6	640.36566	0.321391	11.18	1	10.45	-	+	α-coixin	-
233	RFLPPVSA	8	885.50724	0.317553	10.73	1	7.53	-	+	α-coixin	-
234	VIIPQCSLAPT	11	1197.6427	0.316576	5.06	0	6.19	-	+	α-coixin	-
235	QLLPALSQL	9	981.58588	0.313062	5.48	0	5.54	-	-	α-coixin	-
236	FLPPVSAV	8	828.47454	0.311655	5.56	0	5.26	-	-	α-coixin	-
237	QNQELLPT	8	941.48181	0.309172	3.08	-1	11.81	-	+	α-coixin	-
238	AVASPIASLQ	10	955.53385	0.307975	5.44	0	8.4	-	+	α-coixin	-
239	LTLLCGIHSRSAL	13	1439.7919	0.307606	9.03	1	8.72	-	-	γ-coixin	-
240	QQPIVGGV	8	796.4443	0.307297	5.52	0	9.84	-	-	α-coixin	-
241	LAVASPIASL	10	940.55933	0.307059	5.58	0	6.38	-	-	α-coixin	-
242	VATFLQQQQRQLLPF	15	1815.9996	0.306494	11.11	1	6.82	-	-	α-coixin	-
243	QQQLQQLLPA	10	1165.6455	0.305363	5.49	0	8.64	-	+	α-coixin	-
244	CSLAPSAA	8	775.35344	0.30528	5.23	0	9.19	-	+	α-coixin	-
245	QQLQQQLLPAI	11	1278.7296	0.302054	5.47	0	7.52	-	-	α-coixin	-
246	ADRDRSGIY	9	1051.5047	0.298126	6.88	0	19.08	-	-	γ-coixin	-
247	QQLLASNPL	9	982.54475	0.296391	5.48	0	7.64	-	+	α-coixin	-
248	SILRSSPL	8	871.51272	0.295675	10.85	1	7.61	-	-	α-coixin	-
249	NQILQPY	7	874.45487	0.294731	5.27	0	7.35	-	+	α-coixin	-
250	AVANPITYPA	10	1015.5338	0.294582	5.59	0	8.49	-	+	α-coixin	-
251	FSSNPVSTA	9	908.42396	0.290164	5.53	0	8.85	-	+	α-coixin	-
252	QQILPVLSQL	10	1137.6758	0.288367	5.48	0	5.48	-	+	α-coixin	-
253	CENQILQPY	9	1163.5281	0.286816	3.14	-1	10.96	-	+	α-coixin	-

254	SILQQPLAQL	10	1109.6445	0.284515	5.5	0	6.44	-	-	α-coixin	-
255	QHILPVLSEL	10	1147.6601	0.283339	5.06	-1	9.9	-	+	α-coixin	-
256	QQVLPALSQL	10	1095.6288	0.283033	5.48	0	7.1	-	+	α-coixin	-
257	QQPLIQLQ	8	966.54983	0.282927	5.35	0	7.5	-	+	α-coixin	-
258	RFLPPVSAV	9	984.57565	0.278258	10.73	1	7.07	-	-	α-coixin	-
259	SHRLQ	5	639.34526	0.278044	10.85	1	12.02	-	+	α-coixin	-
260	SAVIIPQ	7	726.42759	0.274376	5.37	0	7.07	-	-	α-coixin	-
261	QLQQILPVLS	10	1137.6758	0.269586	5.37	0	5.48	-	+	α-coixin	-
262	QHQLLQVNPL	10	1188.6615	0.26873	7.59	0	9.32	-	-	α-coixin	-
263	TIAAQQQQQFLPALSQL	17	1884.0105	0.268224	5.47	0	8.29	-	-	α-coixin	-
264	QQPLAQLQQQSSAHLTIQ	18	2018.0545	0.267551	7.59	0	13.06	-	-	α-coixin	-
265	QHILPVLS	8	905.53345	0.265383	7.59	0	7.52	-	+	α-coixin	-
266	SICENQILQPY	11	1363.6442	0.264459	3.14	-1	10.3	-	+	α-coixin	-
267	QILPVLSQL	9	1009.6172	0.264103	5.48	0	4.71	-	+	α-coixin	-
268	LQVNPL	6	682.40138	0.263602	5.58	0	6.7	-	+	α-coixin	-
269	PPVSAI	6	582.33771	0.261988	5.62	0	7.56	-	+	α-coixin	-
270	QNQLLQVNPL	10	1165.6455	0.254267	5.48	0	7.84	-	-	β-coixin	-
271	ICENQILQPY	10	1276.6122	0.250287	3.14	-1	9.84	-	+	α-coixin	-
272	QQLQQLVLP	9	1065.6182	0.248653	5.17	0	6.91	-	+	α-coixin	-
273	IIQRFLPPVSAIGFEHPAVQAY	22	2452.3267	0.247785	7.81	0	11.08	-	-	α-coixin	-
274	GFEHPAVQ	8	883.41882	0.246026	5.06	-1	14.25	-	+	α-coixin	-
275	AVANPITYPAA	11	1086.571	0.24574	5.59	0	8.99	-	-	α-coixin	-
276	LAVASPIASLQ	11	1068.6179	0.245371	5.44	0	7.15	-	+	α-coixin	-
277	AIGFEHPAVQAY	12	1301.6404	0.242867	5.06	-1	13.42	-	+	α-coixin	-
278	QQPIVGAA	8	782.42865	0.241124	5.49	0	10.15	-	-	α-coixin	-
279	QQPLAQLQ	8	924.50288	0.231566	5.35	0	9.12	-	-	α-coixin	-
280	TCLANTARQAFQR	13	1535.7627	0.231049	10.67	2	12.93	-	-	γ-coixin	-
281	LAPTAAIIPQ	10	993.58588	0.22475	5.44	0	7.21	-	+	α-coixin	-
282	HILPVLSEL	9	1019.6015	0.224448	5.06	-1	9.13	-	+	α-coixin	-

283	AIGFEHPAVQ	10	1067.54	0.224209	5.06	-1	13.63	-	+	α-coixin	-
284	GCQPPHLPPPPVHLPPPVHLPPPVHLPPPQ	30	3244.7484	0.223271	7.49	0	15.61	-	-	γ-coixin	-
285	QSLVVIL	7	770.49019	0.222944	5.48	0	4.59	-	+	α-coixin	-
286	SSAHLTIQ	8	855.44503	0.221893	7.63	0	10.3	-	+	α-coixin	-
287	QELLPT	6	699.38031	0.221159	3.08	-1	10.19	-	-	α-coixin	-
288	QQILPVLS	8	896.53312	0.218574	5.37	0	5.96	-	+	α-coixin	-
289	QILPVLS	7	768.47454	0.21638	5.37	0	5.19	-	+	α-coixin	-
290	HQLLQVNPL	9	1060.6029	0.215952	7.69	0	8.55	-	-	α-coixin	-
291	RLQQALAASIL	11	1182.7085	0.213211	10.73	1	8.34	-	-	α-coixin	-
292	IGFEHPAVQ	9	996.50288	0.21196	5.06	-1	13.13	-	+	α-coixin	-
293	LQQILPVLS	9	1009.6172	0.211703	5.45	0	4.71	-	+	α-coixin	-
294	AVASPIA	7	627.35918	0.211456	5.61	0	8.42	-	+	α-coixin	-
295	TIPQYLPSVVATRFEYPVSQSH	22	2518.2856	0.210912	7.53	0	13.13	-	-	α-coixin	-
296	ARVNPA	6	626.35001	0.199462	11.18	1	11.24	-	-	α-coixin	-
297	NQLLQVNPL	9	1037.5869	0.199334	5.36	0	7.07	-	+	β-coixin	-
298	FEHPAVQ	7	826.39735	0.197038	5.06	-1	13.1	-	+	α-coixin	-
299	VASPI	5	485.28495	0.196285	5.57	0	7.42	-	+	α-coixin	-
300	FLPTVSAIGFEHPAVQ	16	1711.8934	0.195859	5.06	-1	11.06	-	-	α-coixin	-
301	ENQILQPY	8	1003.4975	0.193519	3.14	-1	10.98	-	+	α-coixin	-
302	INLQILPQ	8	937.55967	0.188386	5.44	0	5.69	-	+	α-coixin	-
303	NAVYQQQHQLLQVNPL	16	1891.9904	0.188232	7.37	0	11.04	-	-	α-coixin	-
304	VVANPL	6	611.36426	0.185647	5.58	0	7.22	-	+	α-coixin	-
305	TVAFLQ	6	677.37483	0.182791	5.35	0	6	-	+	α-coixin	-
306	FEYPVSQ	7	868.39668	0.181817	3.12	-1	10.02	-	+	α-coixin	-
307	RFEYPVSQ	8	1024.4978	0.180643	6.49	0	11.83	-	+	α-coixin	-
308	VGFEHPAVQ	9	982.48723	0.177278	5.06	-1	13.79	-	+	α-coixin	-
309	AVANPA	6	541.28601	0.176105	5.61	0	9.93	-	+	α-coixin	-
310	EHPAVR	6	707.37147	0.17596	7.76	0	15.85	-	+	α-coixin	-
311	PPVSAV	6	568.32206	0.172618	5.69	0	8.22	-	+	α-coixin	-

312	NAVYQQQHQLL	11	1340.6837	0.171936	7.37	0	10.99	-	-	α-coixin	-
313	VIIPQ	5	568.35845	0.169077	5.44	0	6.11	-	+	α-coixin	-
314	RLQQTLAGSIL	11	1198.7034	0.168165	10.73	1	8.74	-	+	α-coixin	-
315	CSPVTTTIPQYLPSVVATRFEYPVSQ	26	2939.4739	0.157301	6.17	0	10.96	-	+	α-coixin	-
316	QTLAGSIL	8	801.45962	0.155205	5.48	0	7.41	-	-	α-coixin	-
317	NAVYEQQHQLL	11	1341.6677	0.153314	5.06	-1	13.85	-	+	α-coixin	-
318	TSICENQILQPY	12	1464.6919	0.144804	3.14	-1	10.55	-	-	α-coixin	-
319	AVANPNSYLQQQ	12	1331.647	0.141372	5.43	0	11.09	-	-	α-coixin	-
320	AAIIPRFLAPVSAIGFEHPAVQ	22	2303.279	0.141196	7.95	0	12.02	-	-	β-coixin	-
321	AAIIPRFLPPVSAIGFEHPAVQ	22	2329.2947	0.137973	7.95	0	11.66	-	-	α-coixin	-
322	NNAVYEQQHQLL	12	1455.7106	0.137754	5.06	-1	14.7	-	-	α-coixin	-
323	TGFEHPAVQ	9	984.46649	0.133578	5.06	-1	14.5	-	+	α-coixin	-
324	AAIIPRFLPPVSAVGFEHPAVQAY	24	2549.3795	0.13159	7.85	0	12.11	-	-	α-coixin	-
325	SHRLQEALAA	10	1094.5833	0.128898	7.63	0	15.9	-	-	α-coixin	-
326	SSAHLTIQTIAAQQQQQFLPSLS	23	2496.2973	0.127827	7.63	0	11.13	-	-	α-coixin	-
327	TIPQYLPSVVATRFEYPVSQ	20	2294.1947	0.127024	6.52	0	10.34	-	-	α-coixin	-
328	AAIIPRFLPPVSAIGFEHPAVQAY	24	2563.3951	0.120425	7.85	0	11.45	-	-	α-coixin	-
329	IHDKGPTAS	9	924.46649	0.11958	7.87	0	18.05	-	-	γ-coixin	-
330	TATIIPQ	7	742.4225	0.112635	5.35	0	7.57	-	+	α-coixin	-
331	AAIIPQFLPTVSAIGFEHPAVQ	22	2305.2471	0.105346	5.06	-1	10.73	-	-	α-coixin	-
332	AFQRTPDYVEEA	12	1424.6572	0.10445	3.74	-2	19.89	-	-	γ-coixin	-
333	EHPAVQ	6	679.32894	0.0993281	5.06	-1	14.81	-	+	α-coixin	-
334	EHPAVQA	7	750.36605	0.0974816	5.06	-1	15.31	-	+	α-coixin	-
335	NAVYEQQHQLLQ	12	1469.7263	0.0945529	5.06	-1	14.62	-	-	α-coixin	-
336	EYPVSQ	6	721.32827	0.0835248	3.12	-1	11.73	-	+	α-coixin	-
337	TPDYVEE	7	851.35488	0.0757859	2.79	-3	18.02	-	+	γ-coixin	-
338	TPDYVEEA	8	922.39199	0.0724375	2.91	-3	18.52	-	+	γ-coixin	-
339	QRTPDYVEEA	10	1206.5517	0.0699371	3.74	-2	21.1	-	+	γ-coixin	-
340	QRTPDYVEE	9	1135.5146	0.0697131	3.73	-2	20.6	-	-	γ-coixin	-

341	VVANPAAYLQQQLFSSNPVSTA	22	2304.175	0.0628216	5.58	0	9.52	-	+	*α*-coixin	-
342	RTPDYVEEA	9	1078.4931	0.0619354	3.74	-2	20.33	-	-	*γ*-coixin	-
343	QEAIAE	6	659.31262	0.0602902	2.92	-2	15.81	-	+	*α*-coixin	-
