# Supplementary material for: Screening and Activity Analysis of α-Glucosidase Inhibitory Peptides Derived from Coix Seed Prolamins Using Bioinformatics and Molecular Docking
Source: Foods. 2023 Oct 30;12(21):3970. doi: 10.3390/foods12213970 (PMC10649794; doi:10.3390/foods12213970)
Supplement: Supplementary file 1 [file foods-12-03970-s001.zip › Figure S2. MS spectrum.pdf]

LFPSNPLA

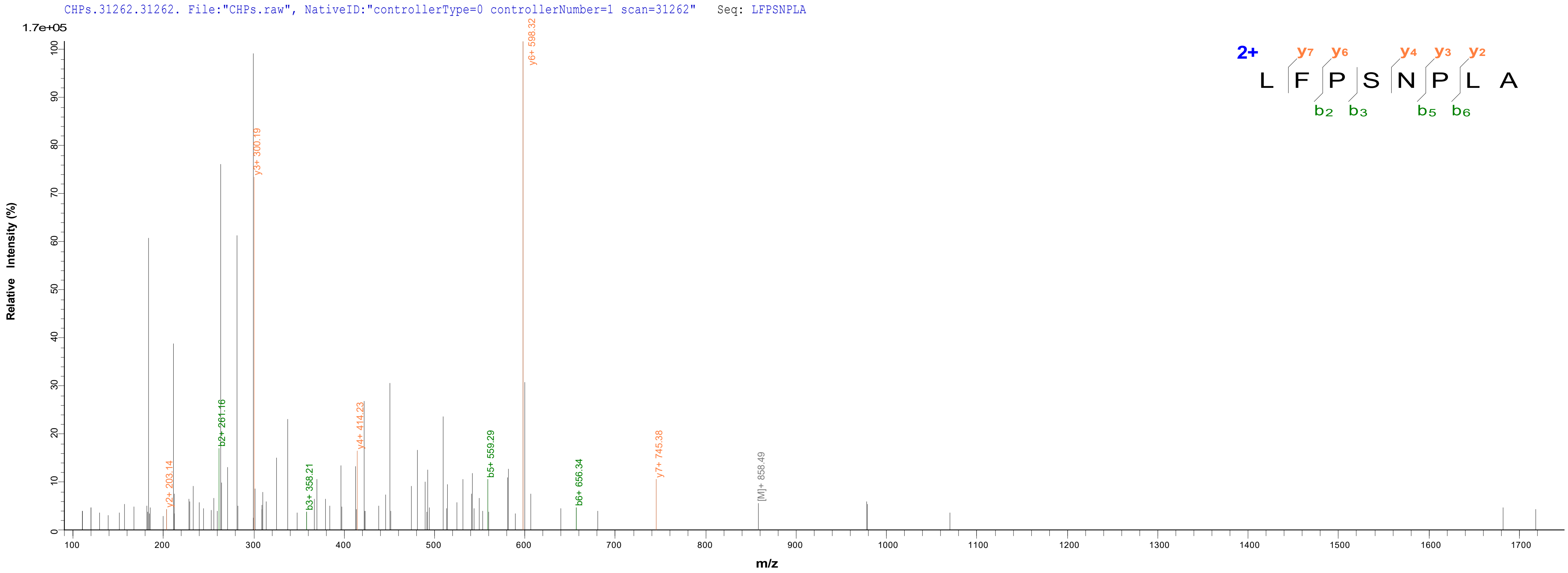

CHPs.31262.31262. File:"CHPs.raw", NativeID:"controllerType=0 controllerNumber=1 scan=31262" Seq: LFPSNPLA
1.7e+05
Relative Intensity (%)
m/z
y6+ 598.32
y3+ 300.19
b2+ 261.16
y4+ 414.23
b5+ 559.29
y7+ 745.38
[M]+ 858.49
y2+ 203.14
b3+ 358.21
b6+ 656.34
2+
L F P S N P L A
y7 y6 y4 y3 y2
b2 b3 b5 b6

FPCNPLVA

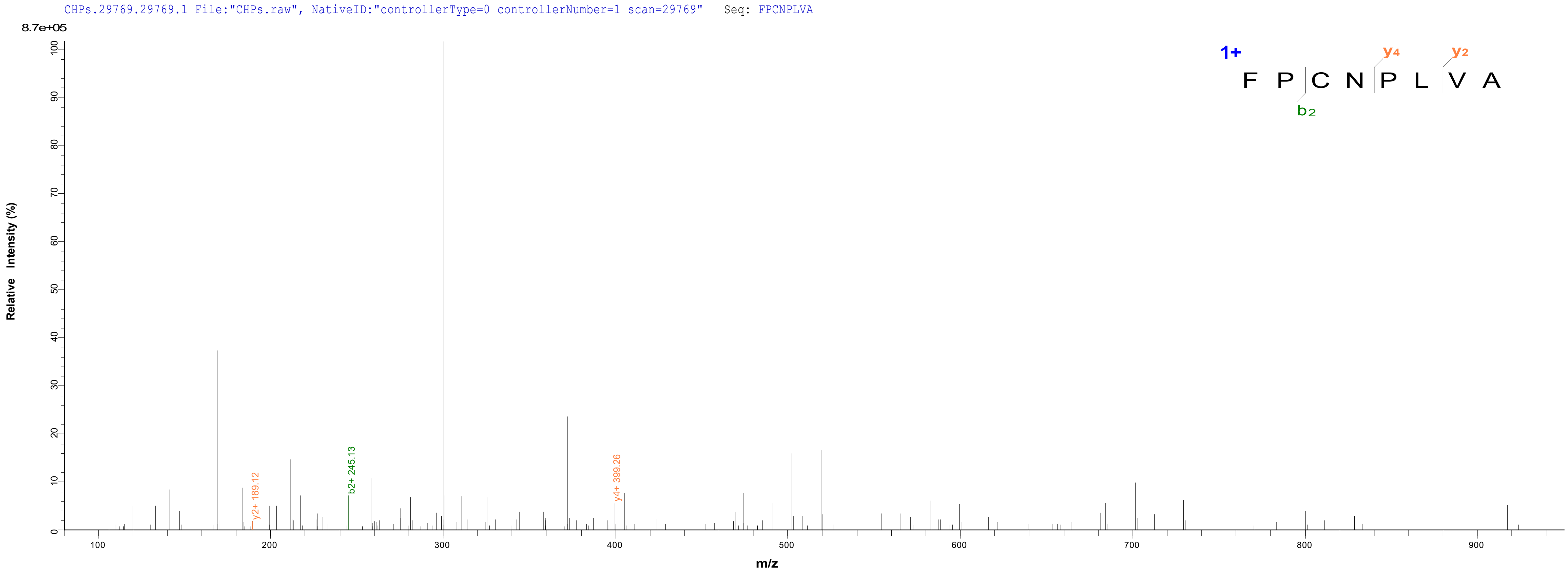
CHPs.29769.29769.1 File:"CHPs.raw", NativeID:"controllerType=0 controllerNumber=1 scan=29769" Seq: FPCNPLVA
8.7e+05
Relative Intensity (%)
m/z
y2+ 189.12
b2+ 245.13
y4+ 399.26
1+
F P C N P L V A
y4
y2
b2

FPCNPLVA

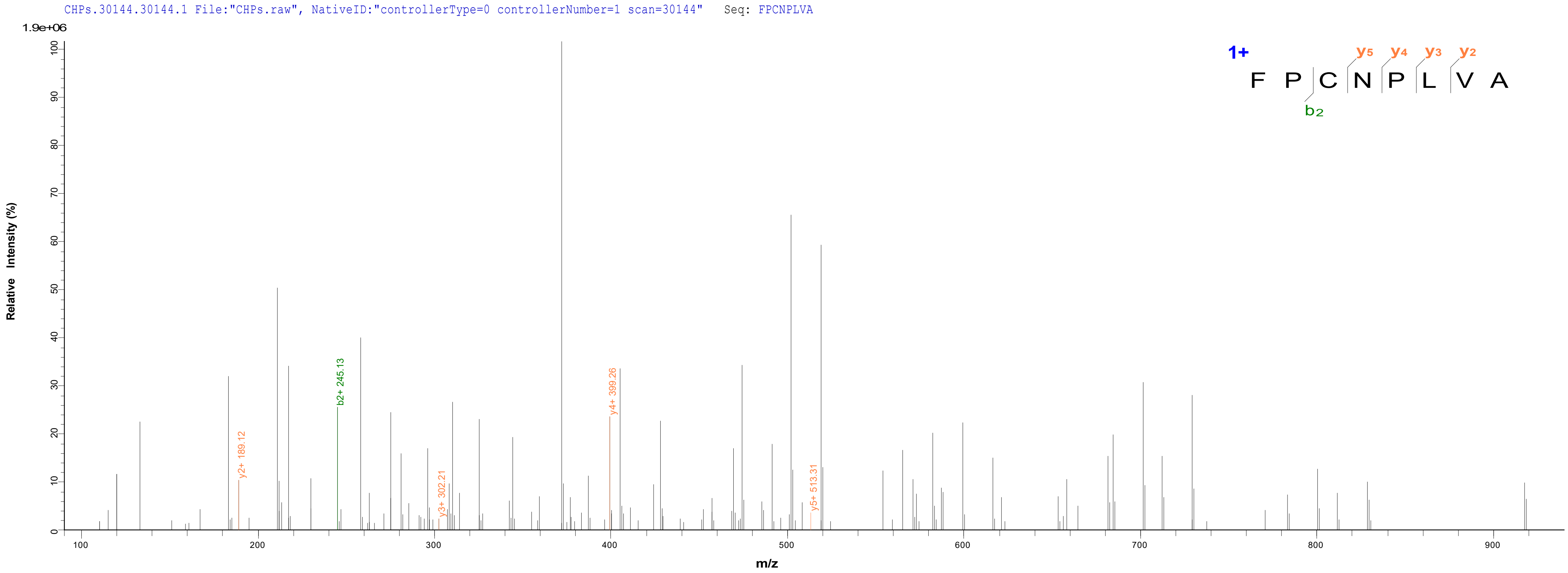
CHPs.30144.30144.1 File:"CHPs.raw", NativeID:"controllerType=0 controllerNumber=1 scan=30144" Seq: FPCNPLVA
1.9e+06
Relative Intensity (%)
m/z
y2+ 189.12
b2+ 245.13
y3+ 302.21
y4+ 399.26
y5+ 513.31
1+
F P C N P L V A
y5 y4 y3 y2
b2

HLPFNPQ

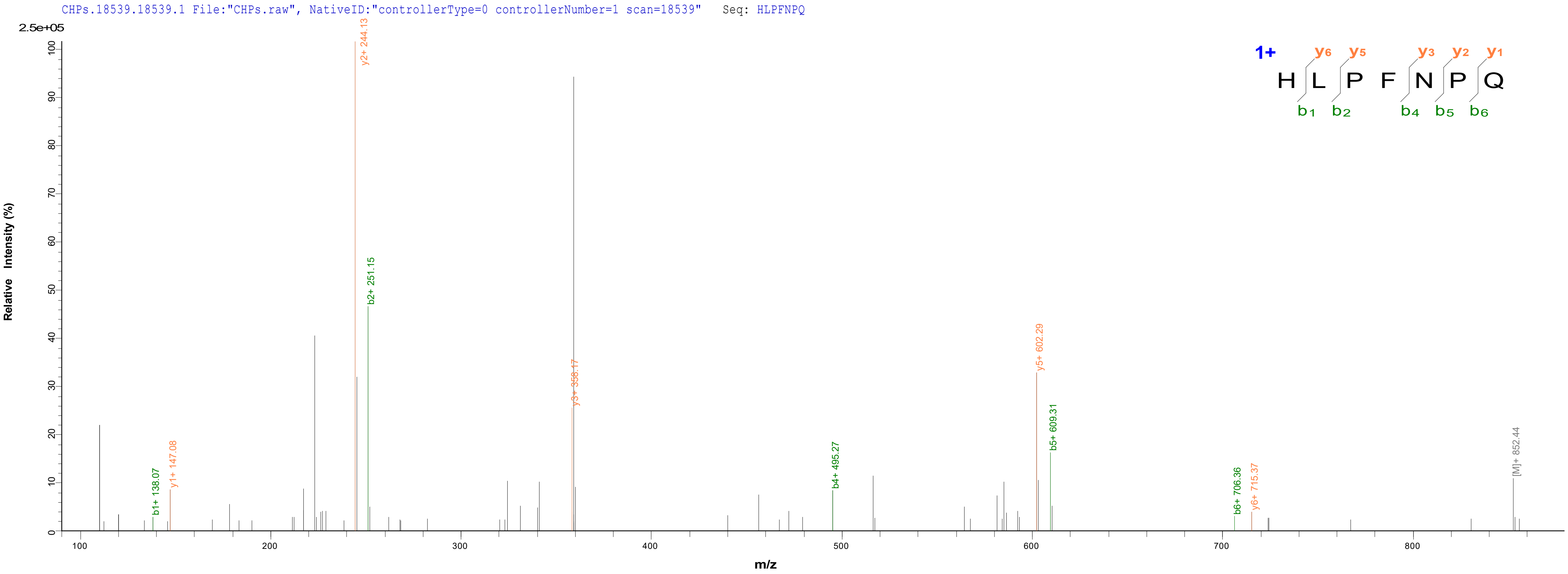

CHPs.18539.18539.1 File:"CHPs.raw", NativeID:"controllerType=0 controllerNumber=1 scan=18539" Seq: HLPFNPQ
2.5e+05
Relative Intensity (%)
m/z
1+
H L P F N P Q
y6 y5 y3 y2 y1
b1 b2 b4 b5 b6
y2+ 244.13
b2+ 251.15
y3+ 358.17
y5+ 602.29
b5+ 609.31
b1+ 138.07
y1+ 147.08
b4+ 495.27
b6+ 706.36
y6+ 715.37
[M]+ 852.44

HLPFNPQ

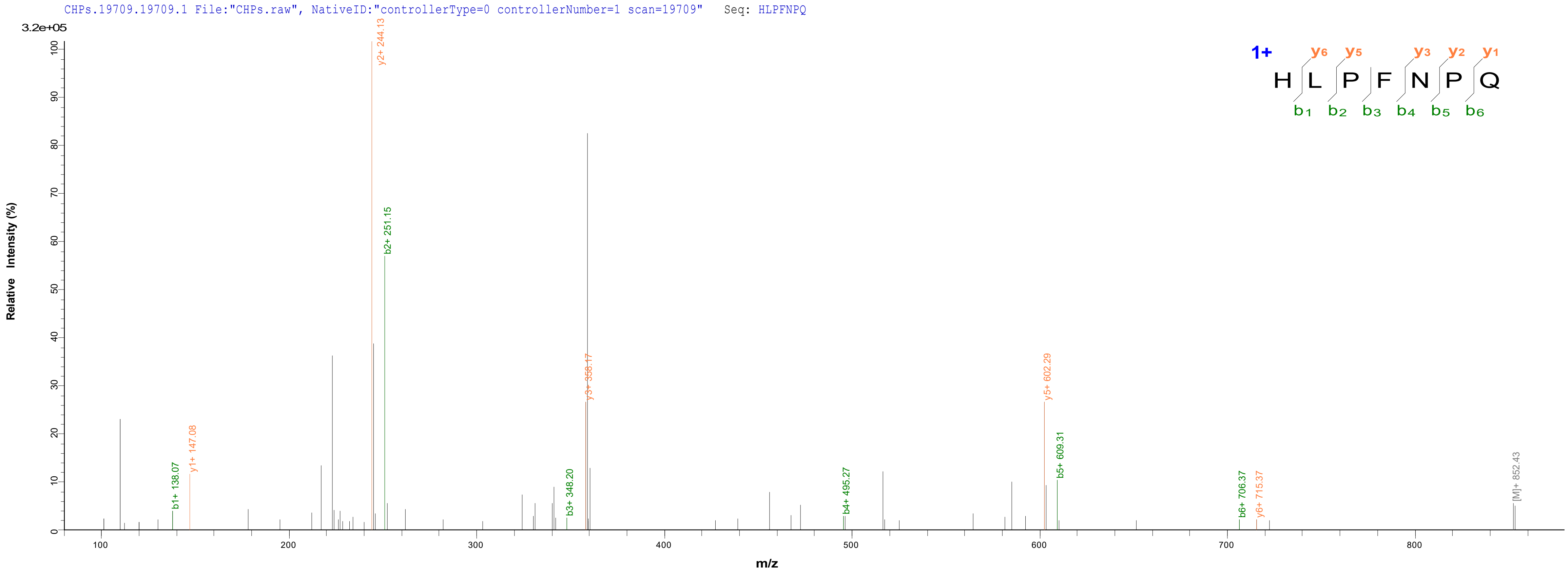

CHPs.19709.19709.1 File:"CHPs.raw", NativeID:"controllerType=0 controllerNumber=1 scan=19709" Seq: HLPFNPQ
3.2e+05
Relative Intensity (%)
m/z
y2+ 244.13
b2+ 251.15
y3+ 358.17
y5+ 602.29
b5+ 609.31
y1+ 147.08
b1+ 138.07
b3+ 348.20
b4+ 495.27
b6+ 706.37
y6+ 715.37
[M]+ 852.43
1+
H L P F N P Q
y6 y5 y3 y2 y1
b1 b2 b3 b4 b5 b6

HLPFNPQ

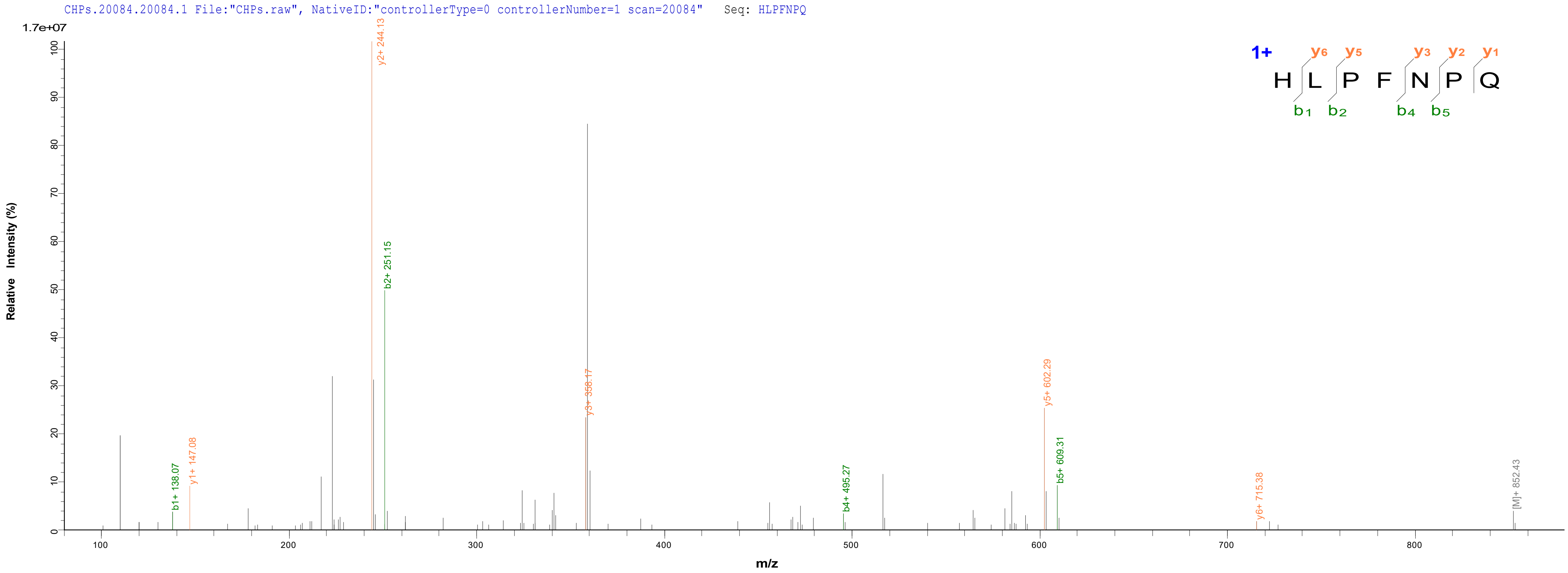

CHPs.20084.20084.1 File:"CHPs.raw", NativeID:"controllerType=0 controllerNumber=1 scan=20084" Seq: HLPFNPQ
1.7e+07
Relative Intensity (%)
m/z
y2+ 244.13
b2+ 251.15
y3+ 358.17
y5+ 602.29
b5+ 609.31
b1+ 138.07
y1+ 147.08
b4+ 495.27
y6+ 715.38
[M]+ 852.43
1+
H L P F N P Q
y6 y5 y3 y2 y1
b1 b2 b4 b5

LLPFYPN

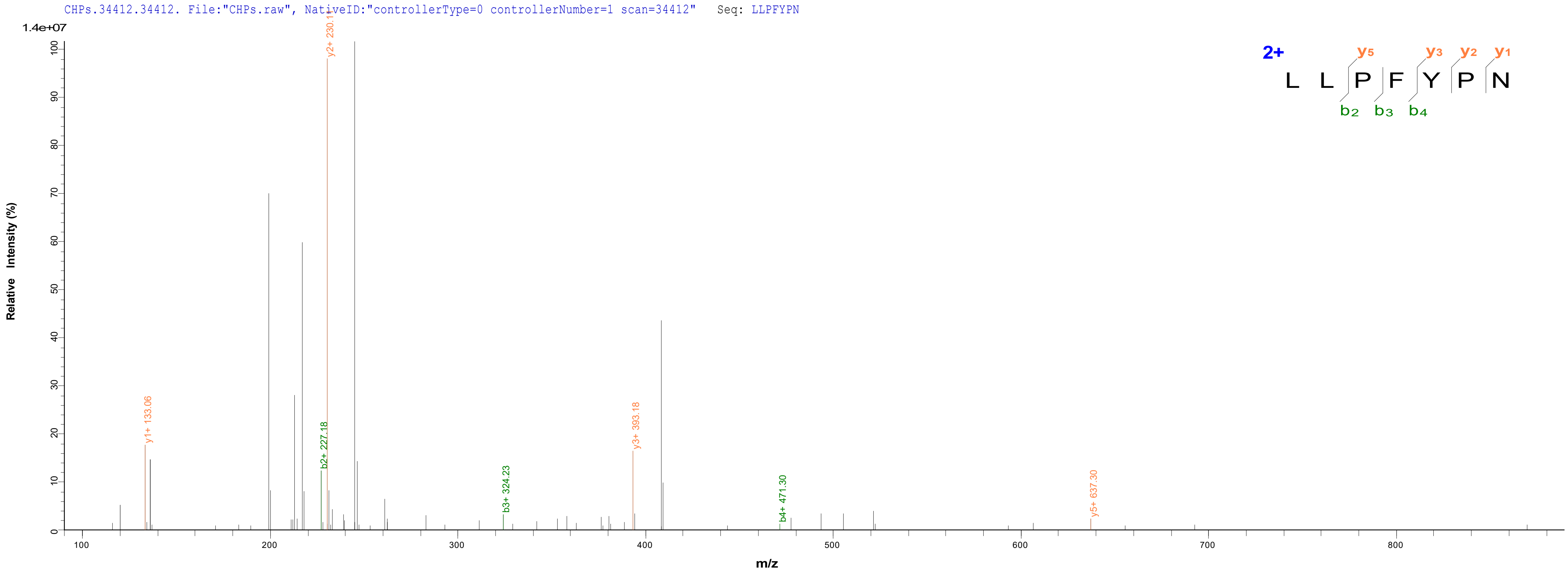
CHPs.34412.34412. File:"CHPs.raw", NativeID:"controllerType=0 controllerNumber=1 scan=34412" Seq: LLPFYPN
1.4e+07
Relative Intensity (%)
m/z
y1+ 133.06
b2+ 227.18
y2+ 230.1
b3+ 324.23
y3+ 393.18
b4+ 471.30
y5+ 637.30
2+
L L P F Y P N
y5 y3 y2 y1
b2 b3 b4

LLPFYPN

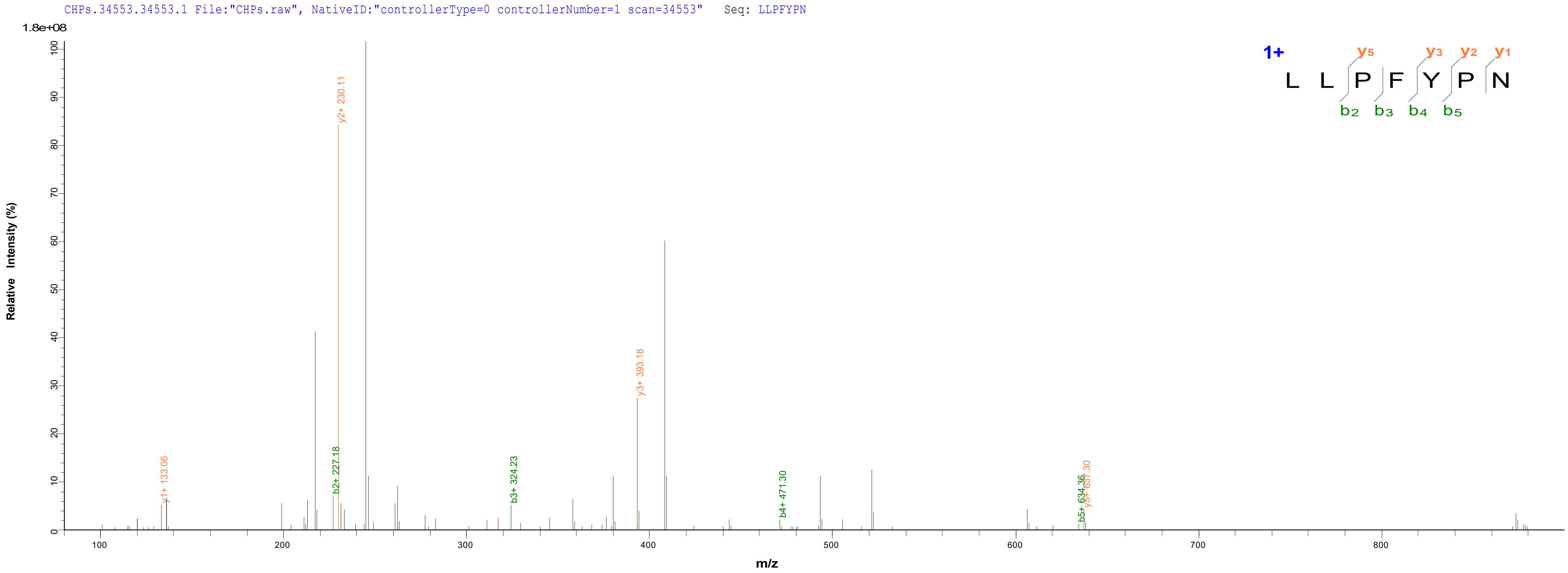
CHPs.34553.34553.1 File:"CHPs.raw", NativeID:"controllerType=0 controllerNumber=1 scan=34553" Seq: LLPFYPN
1.8e+08
Relative Intensity (%)
m/z
y1+ 133.06
b2+ 227.18
y2+ 230.11
b3+ 324.23
y3+ 393.18
b4+ 471.30
b5+ 634.36
y5+ 637.30
1+
L L P F Y P N
y5 y3 y2 y1
b2 b3 b4 b5

LLPFYPN

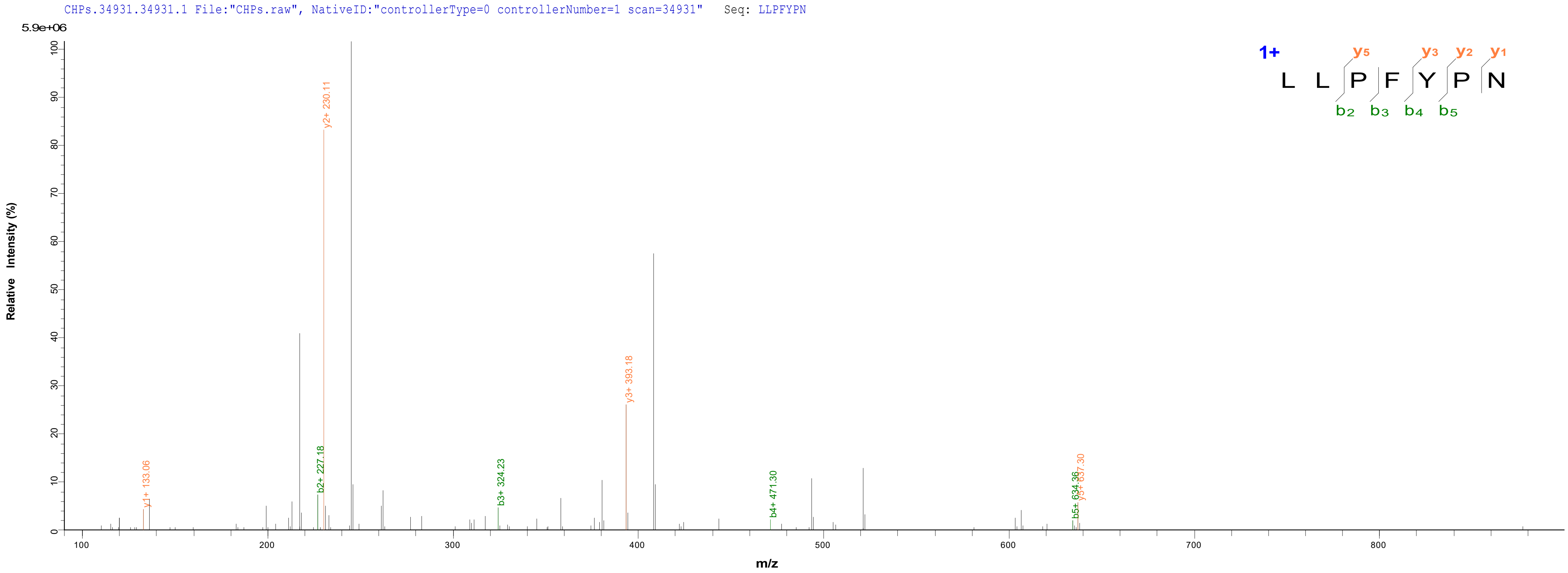

CHPs.34931.34931.1 File:"CHPs.raw", NativeID:"controllerType=0 controllerNumber=1 scan=34931" Seq: LLPFYPN
5.9e+06
Relative Intensity (%)
m/z
y1+ 133.06
b2+ 227.18
y2+ 230.11
b3+ 324.23
y3+ 393.18
b4+ 471.30
b5+ 634.36
y5+ 637.30
1+
L L P F Y P N
y5 y3 y2 y1
b2 b3 b4 b5

FLSPF

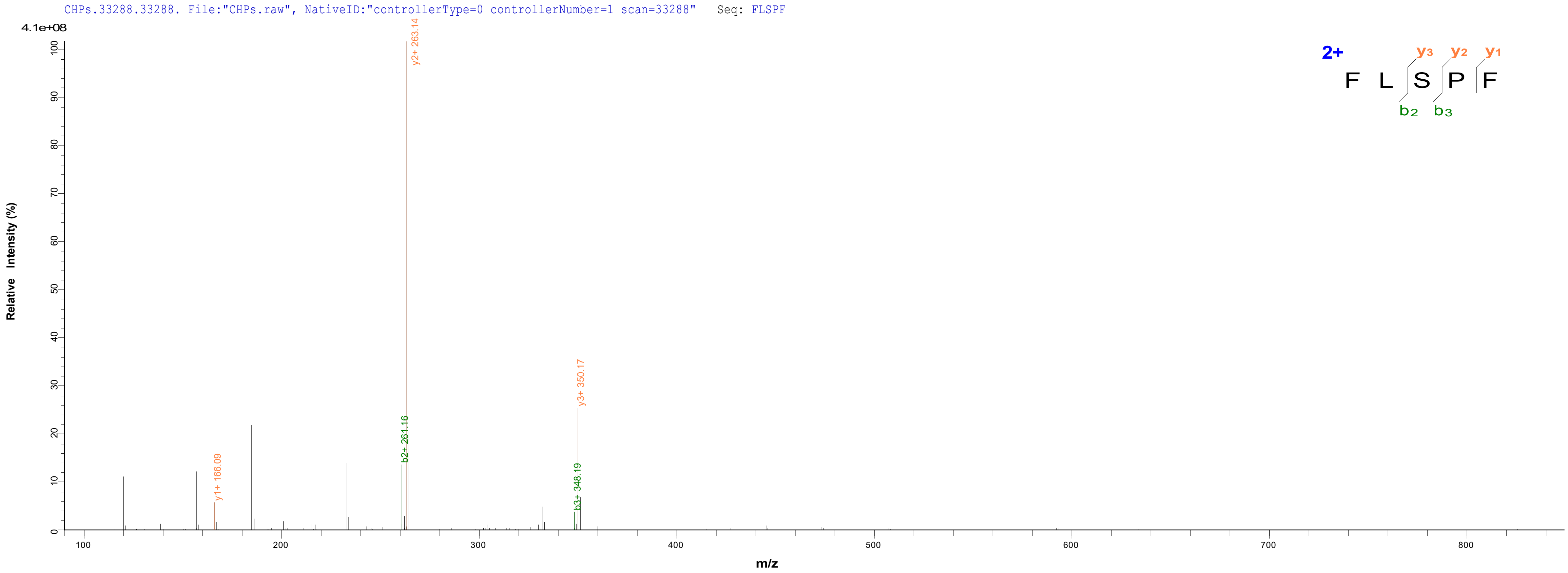
CHPs.33288.33288. File:"CHPs.raw", NativeID:"controllerType=0 controllerNumber=1 scan=33288" Seq: FLSPF
4.1e+08
Relative Intensity (%)
m/z
y2+ 263.14
y3+ 350.17
b2+ 261.16
y1+ 166.09
b3+ 348.19
2+
F L S P F
y3 y2 y1
b2 b3

FPCNPLVA

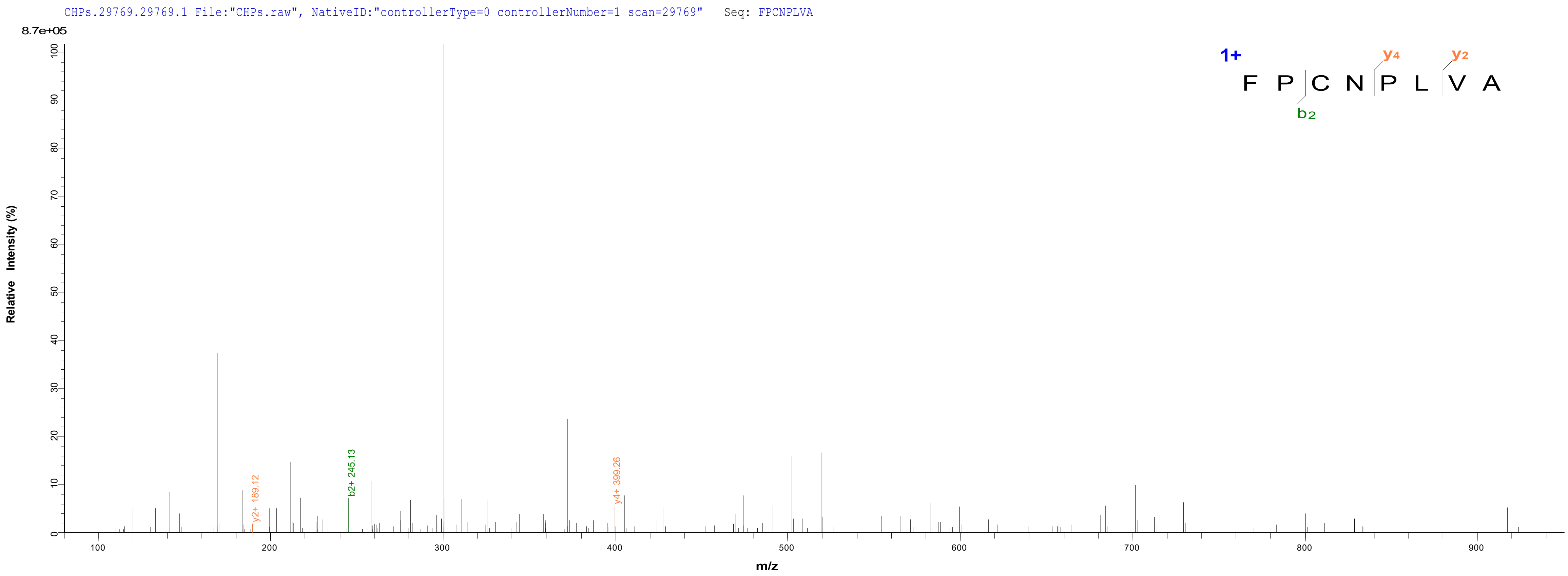
CHPs.29769.29769.1 File:"CHPs.raw", NativeID:"controllerType=0 controllerNumber=1 scan=29769" Seq: FPCNPLVA
8.7e+05
Relative Intensity (%)
m/z
1+
F P C N P L V A
y4
y2
b2
y2+ 189.12
b2+ 245.13
y4+ 399.26

FPCNPLVA

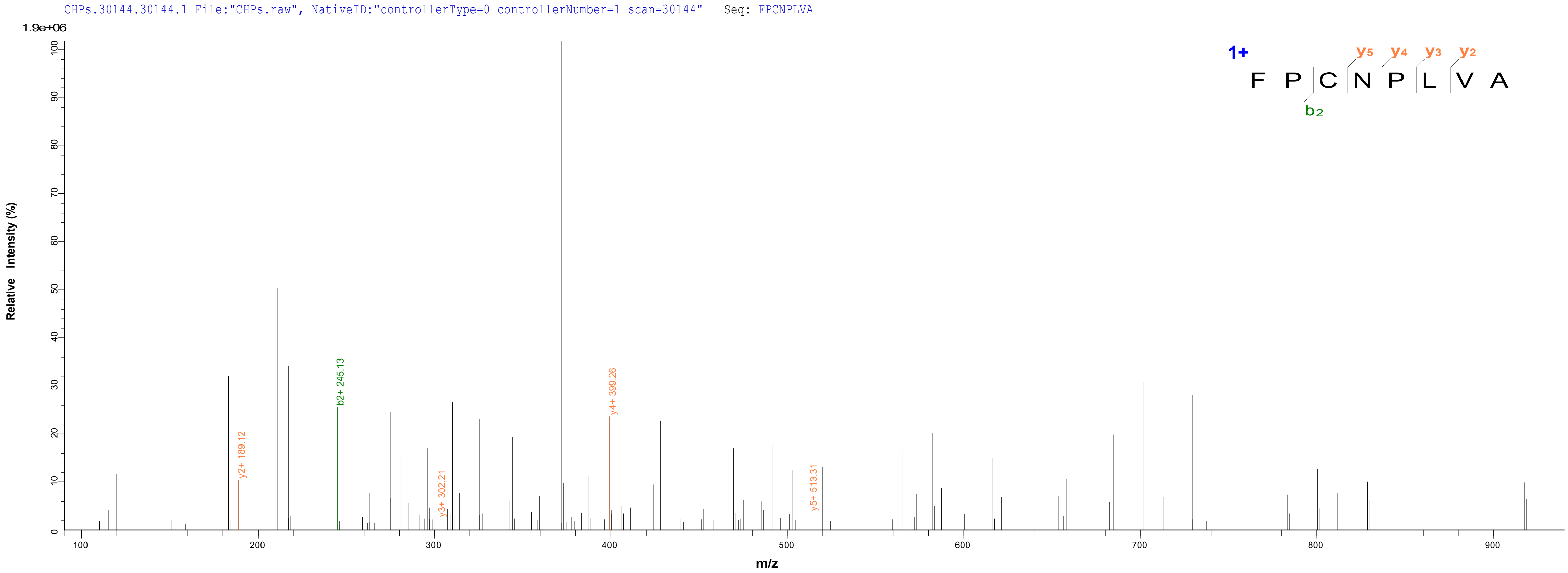
CHPs.30144.30144.1 File:"CHPs.raw", NativeID:"controllerType=0 controllerNumber=1 scan=30144" Seq: FPCNPLVA
1.9e+06
Relative Intensity (%)
m/z
1+
F P C N P L V A
y5 y4 y3 y2
b2
y2+ 189.12
b2+ 245.13
y3+ 302.21
y4+ 399.26
y5+ 513.31

LPPFLPS

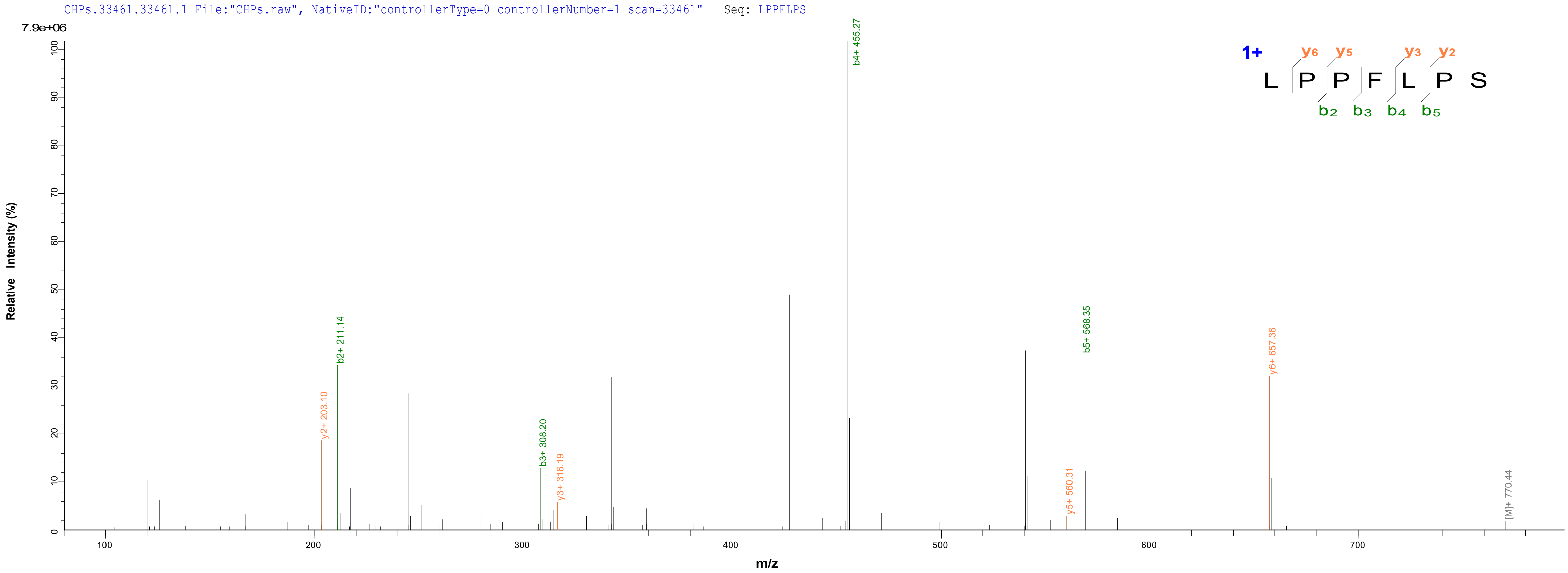

CHPs.33461.33461.1 File:"CHPs.raw", NativeID:"controllerType=0 controllerNumber=1 scan=33461" Seq: LPPFLPS
7.9e+06
Relative Intensity (%)
m/z
b4+ 455.27
b2+ 211.14
y2+ 203.10
b3+ 308.20
y3+ 316.19
y5+ 560.31
b5+ 568.35
y6+ 657.36
[M]+ 770.44
1+
L P P F L P S
y6 y5 y3 y2
b2 b3 b4 b5

QLFPSNPLA

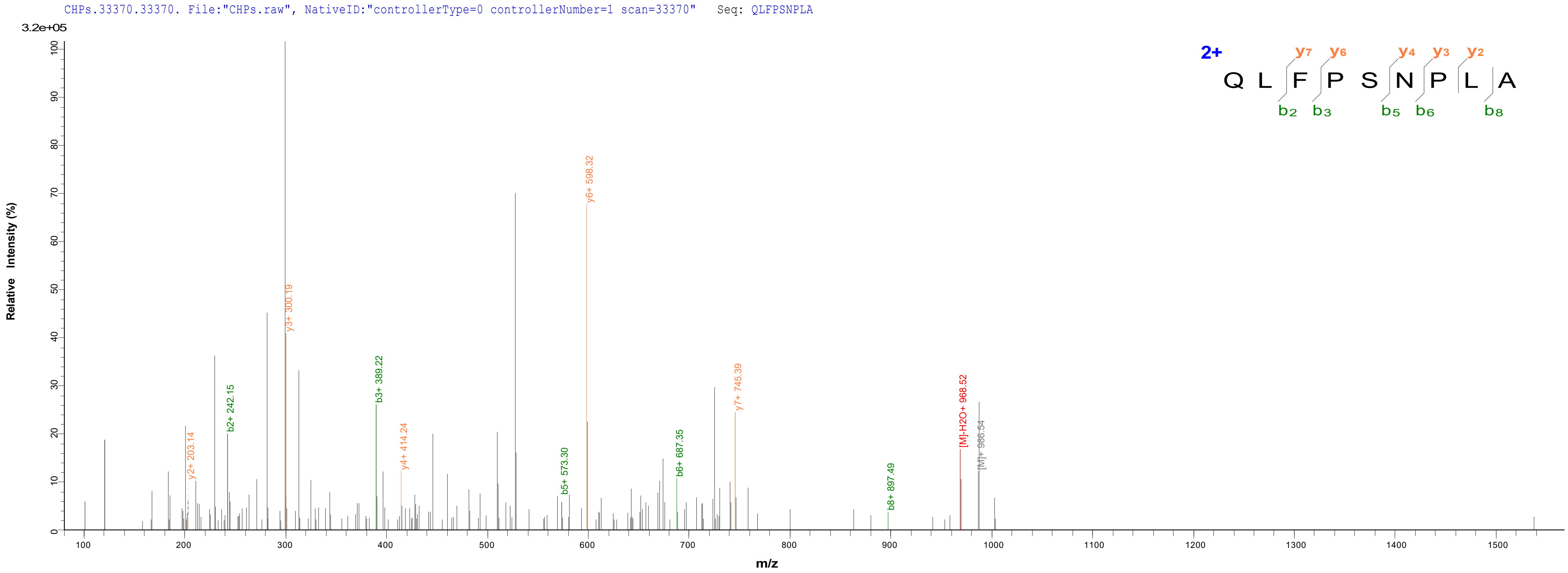

CHPs.33370.33370. File:"CHPs.raw", NativeID:"controllerType=0 controllerNumber=1 scan=33370" Seq: QLFPSNPLA
3.2e+05
Relative Intensity (%)
m/z
y2+ 203.14
b2+ 242.15
y3+ 300.19
b3+ 389.22
y4+ 414.24
b5+ 573.30
y6+ 598.32
b6+ 687.35
y7+ 745.39
b8+ 897.49
[M]-H2O+ 968.52
[M]+ 986.54
2+
Q L F P S N P L A
y7 y6 y4 y3 y2
b2 b3 b5 b6 b8

QQHLPFNPQ

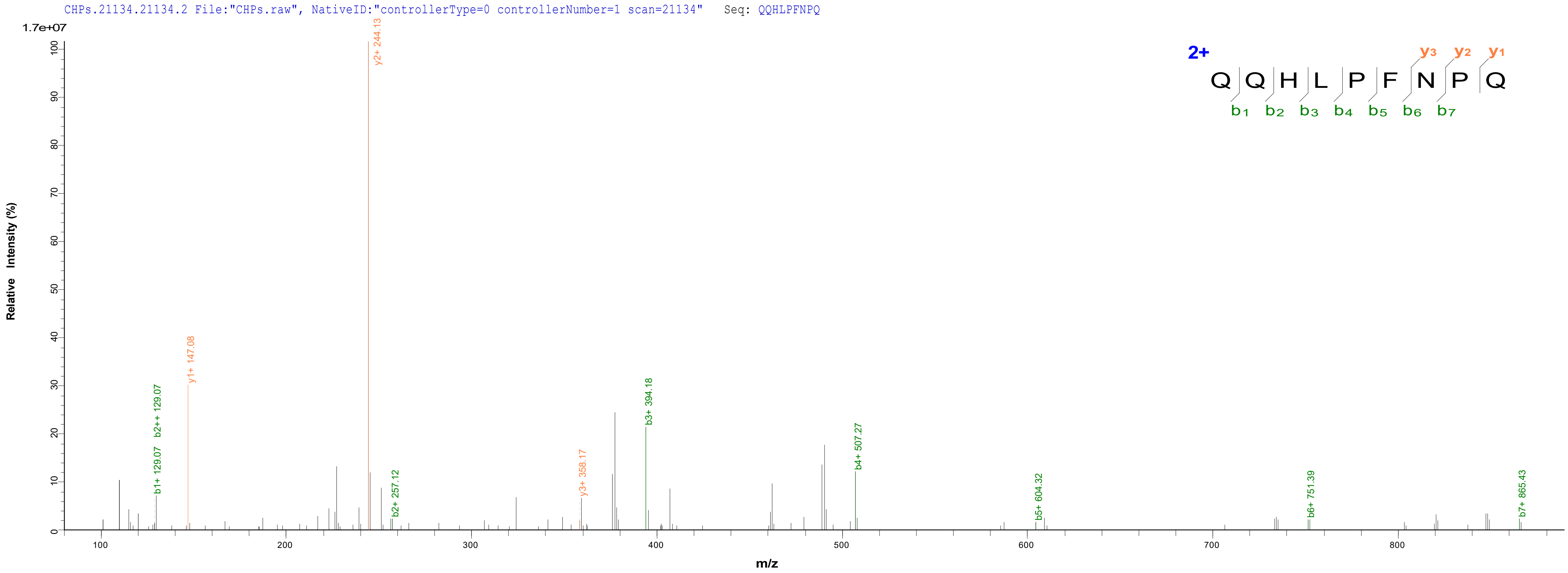

CHPs.21134.21134.2 File:"CHPs.raw", NativeID:"controllerType=0 controllerNumber=1 scan=21134" Seq: QQHLPFNPQ
1.7e+07
Relative Intensity (%)
m/z
y2+ 244.13
y1+ 147.08
b2++ 129.07
b1+ 129.07
b2+ 257.12
y3+ 358.17
b3+ 394.18
b4+ 507.27
b5+ 604.32
b6+ 751.39
b7+ 865.43
2+
Q Q H L P F N P Q
y3 y2 y1
b1 b2 b3 b4 b5 b6 b7

QQHLPFNPQ

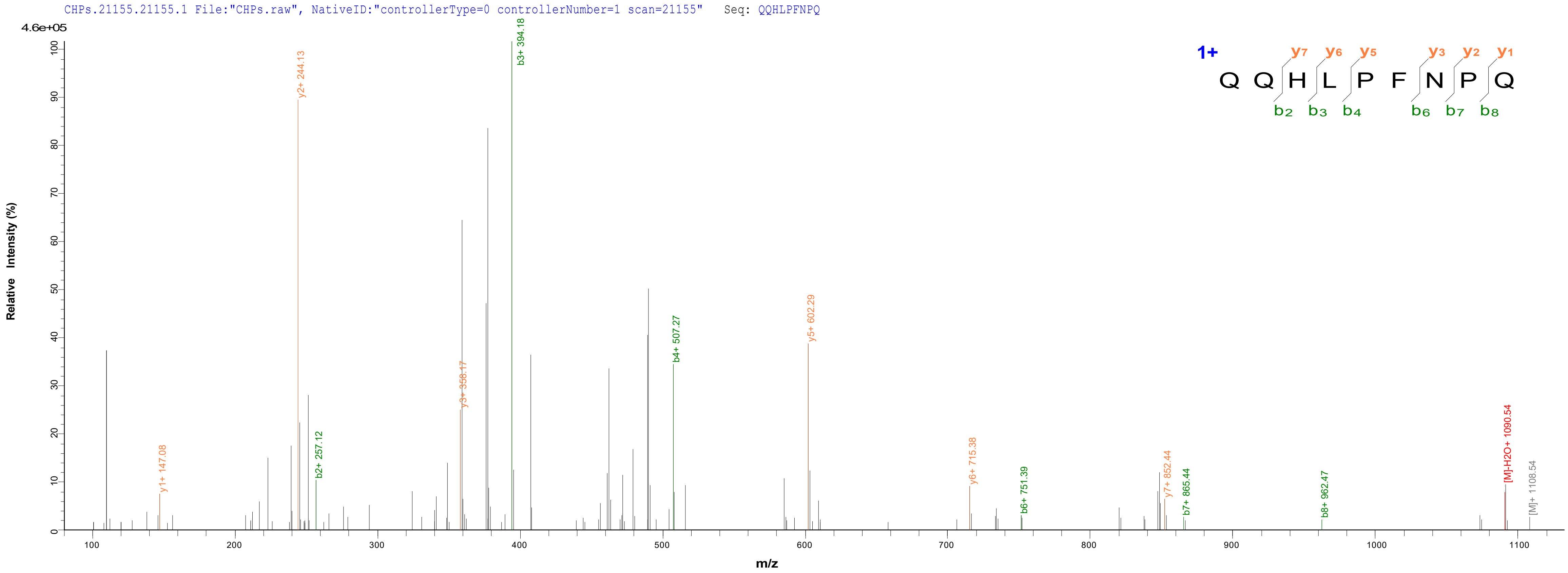

CHPs.21155.21155.1 File:"CHPs.raw", NativeID:"controllerType=0 controllerNumber=1 scan=21155" Seq: QQHLPFNPQ
4.6e+05
Relative Intensity (%)
m/z
1+
Q Q H L P F N P Q
y7 y6 y5 y3 y2 y1
b2 b3 b4 b6 b7 b8
y1+ 147.08
y2+ 244.13
b2+ 257.12
y3+ 358.17
b3+ 394.18
b4+ 507.27
y5+ 602.29
y6+ 715.38
b6+ 751.39
y7+ 852.44
b7+ 865.44
b8+ 962.47
[M]-H2O+ 1090.54
[M]+ 1108.54

SWQQPIVGR

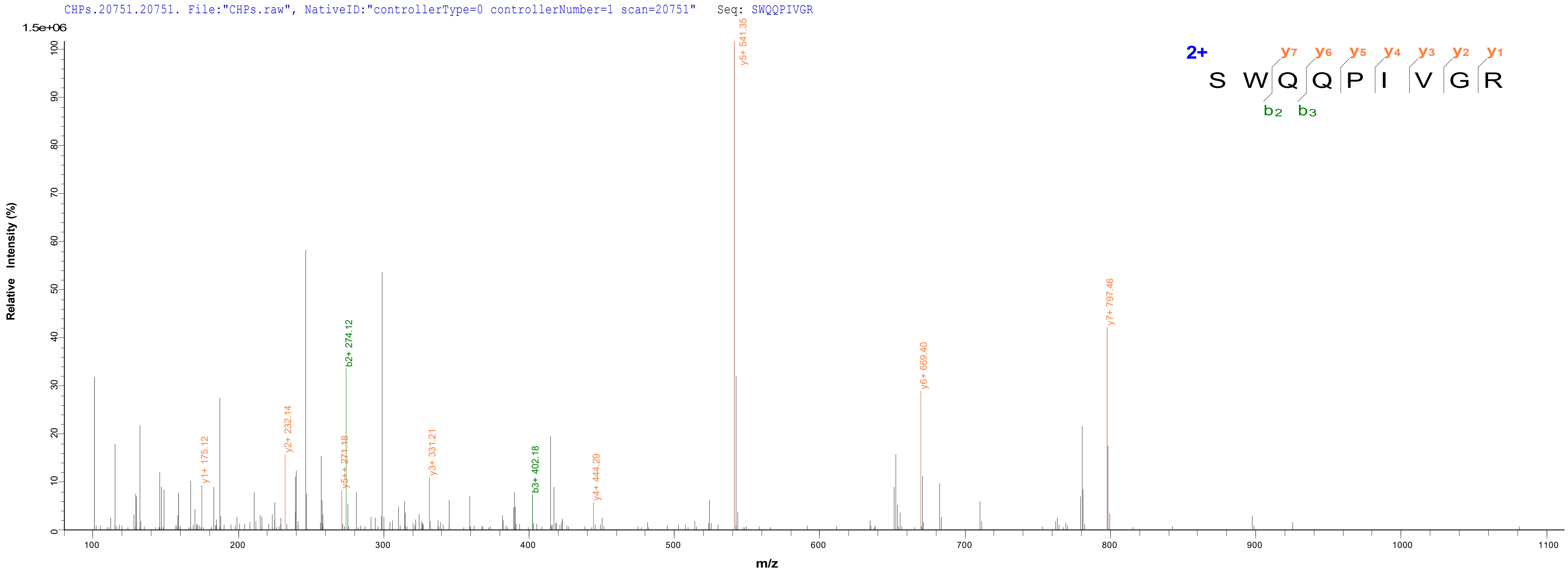
CHPs.20751.20751. File:"CHPs.raw", NativeID:"controllerType=0 controllerNumber=1 scan=20751" Seq: SWQQPIVGR
1.5e+06
Relative Intensity (%)
m/z
y5+ 541.35
y7+ 797.46
y6+ 669.40
b2+ 274.12
y2+ 232.14
y1+ 175.12
y5++ 271.18
y3+ 331.21
b3+ 402.18
y4+ 444.29
2+
S W Q Q P I V G R
y7 y6 y5 y4 y3 y2 y1
b2 b3

SWQQPIVGR

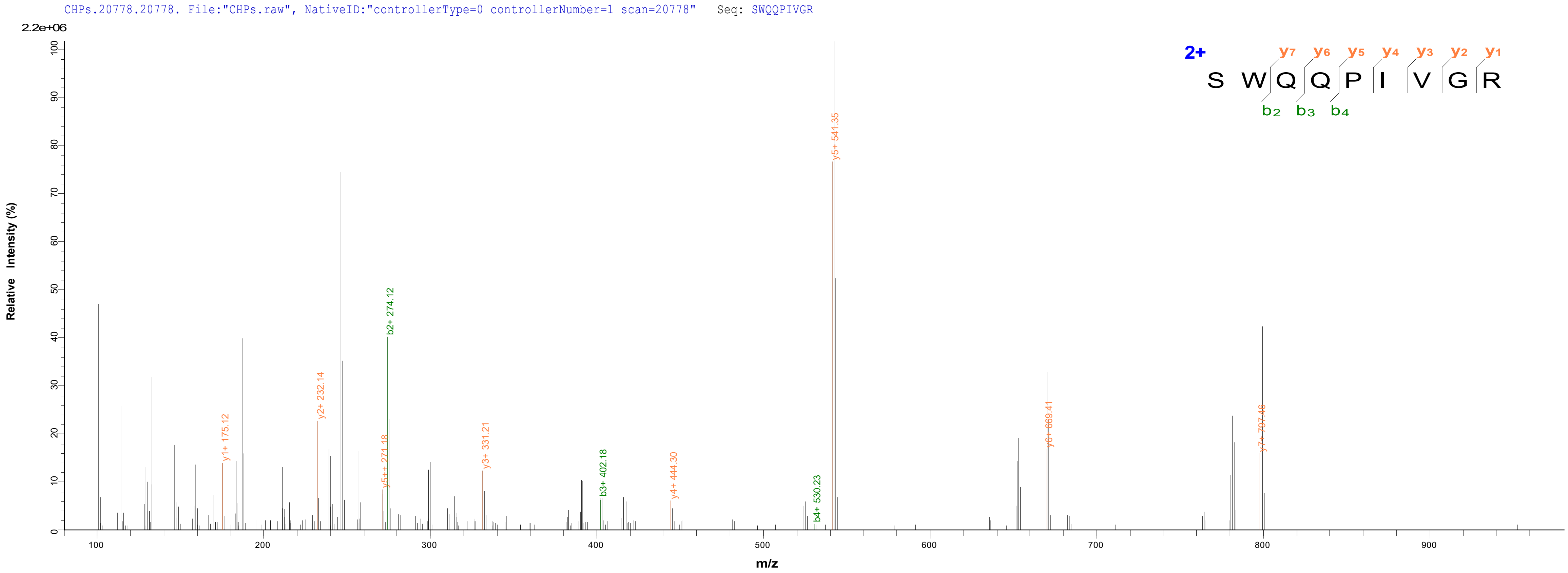

CHPs.20778.20778. File:"CHPs.raw", NativeID:"controllerType=0 controllerNumber=1 scan=20778" Seq: SWQQPIVGR
2.2e+06
Relative Intensity (%)
m/z
2+
S W Q Q P I V G R
y7 y6 y5 y4 y3 y2 y1
b2 b3 b4
y1+ 175.12
y2+ 232.14
y5++ 271.18
b2+ 274.12
y3+ 331.21
b3+ 402.18
y4+ 444.30
b4+ 530.23
y5+ 541.35
y6+ 669.41
y7+ 797.40

SWQQPIVGR

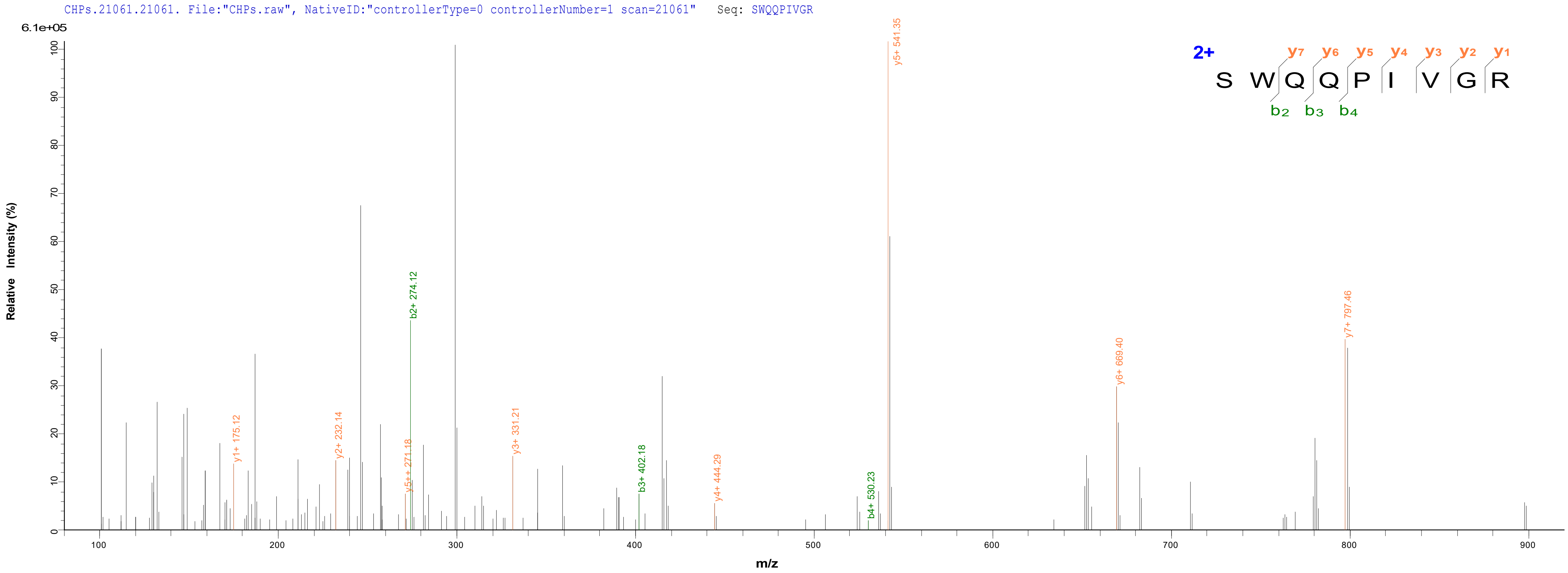

CHPs.21061.21061. File:"CHPs.raw", NativeID:"controllerType=0 controllerNumber=1 scan=21061" Seq: SWQQPIVGR
6.1e+05
Relative Intensity (%)
m/z
2+
S W Q Q P I V G R
y7 y6 y5 y4 y3 y2 y1
b2 b3 b4
y5+ 541.35
y7+ 797.46
y6+ 669.40
b2+ 274.12
y3+ 331.21
y2+ 232.14
y1+ 175.12
y5++ 271.18
b3+ 402.18
y4+ 444.29
b4+ 530.23

SWQQPIVGR

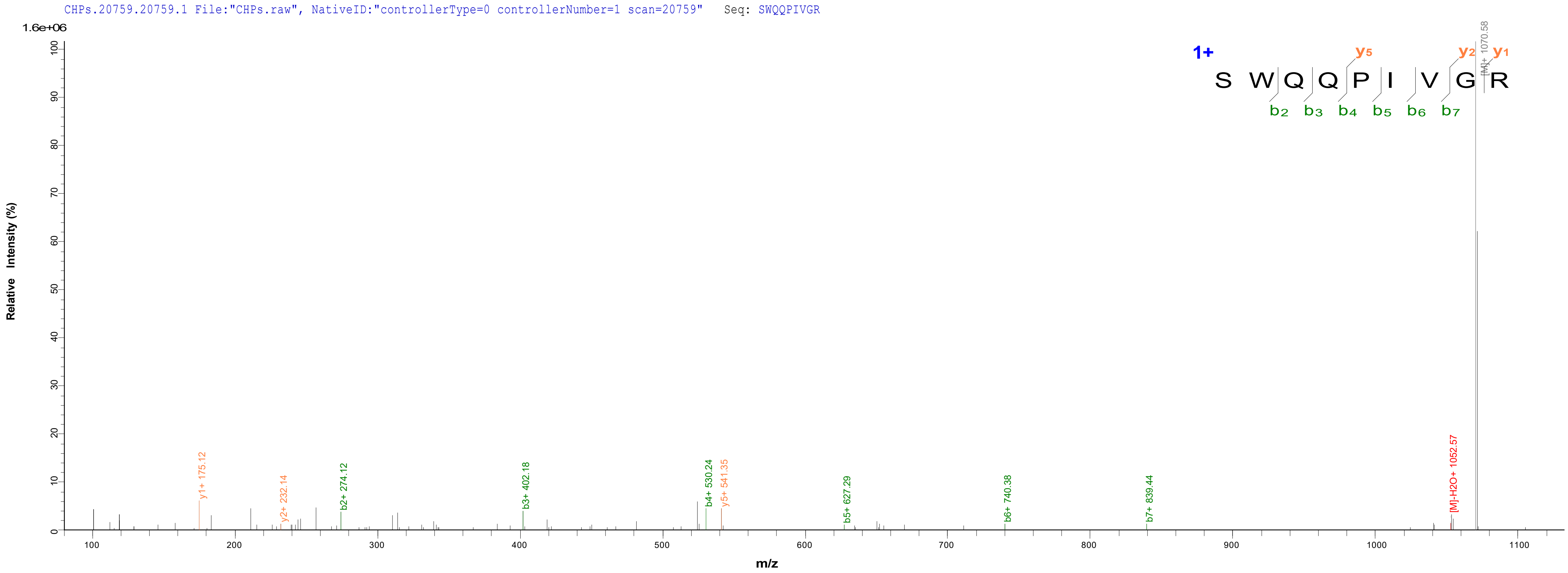
CHPs.20759.20759.1 File:"CHPs.raw", NativeID:"controllerType=0 controllerNumber=1 scan=20759" Seq: SWQQPIVGR
1.6e+06
Relative Intensity (%)
m/z
1+
S W Q Q P I V G R
y5 y2 y1
b2 b3 b4 b5 b6 b7
y1+ 175.12
y2+ 232.14
b2+ 274.12
b3+ 402.18
b4+ 530.24
y5+ 541.35
b5+ 627.29
b6+ 740.38
b7+ 839.44
[M]-H2O+ 1052.57
[M]+ 1070.58

SWQQPIVGR

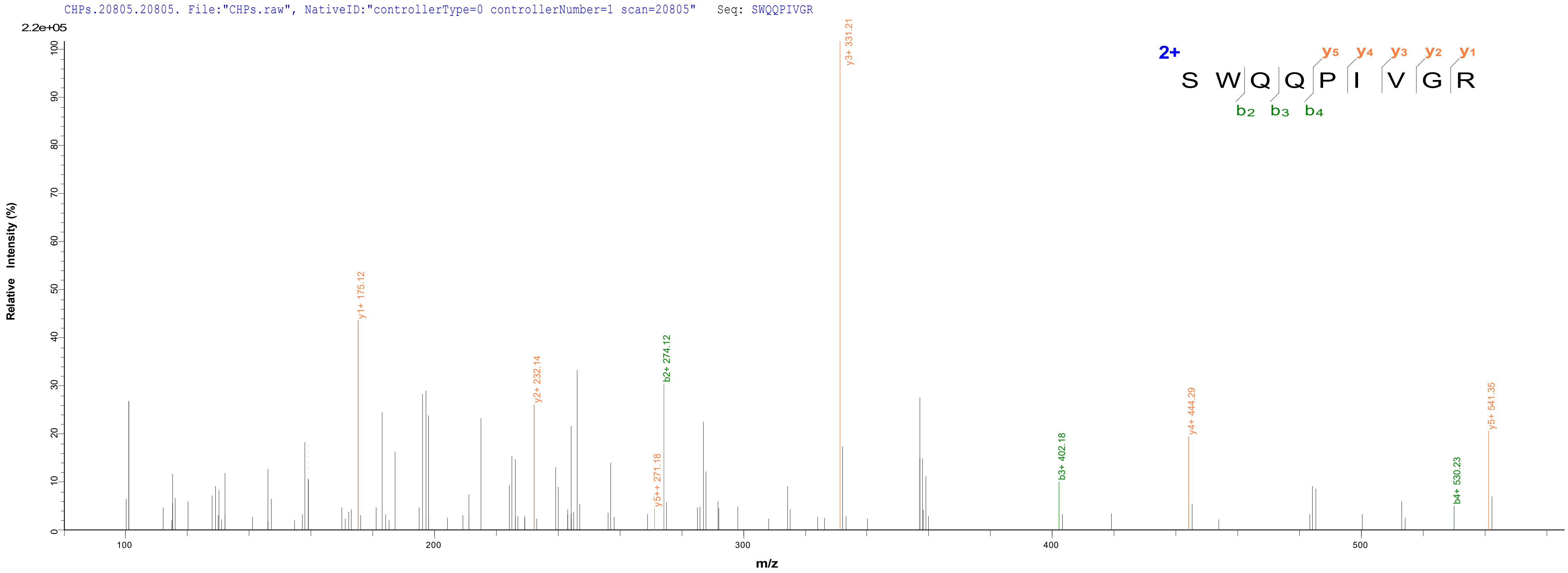
CHPs.20805.20805. File:"CHPs.raw", NativeID:"controllerType=0 controllerNumber=1 scan=20805" Seq: SWQQPIVGR
2.2e+05
Relative Intensity (%)
m/z
2+
S W Q Q P I V G R
y5 y4 y3 y2 y1
b2 b3 b4
y1+ 175.12
y2+ 232.14
y5++ 271.18
b2+ 274.12
y3+ 331.21
b3+ 402.18
y4+ 444.29
b4+ 530.23
y5+ 541.35

SWQQPIVGR

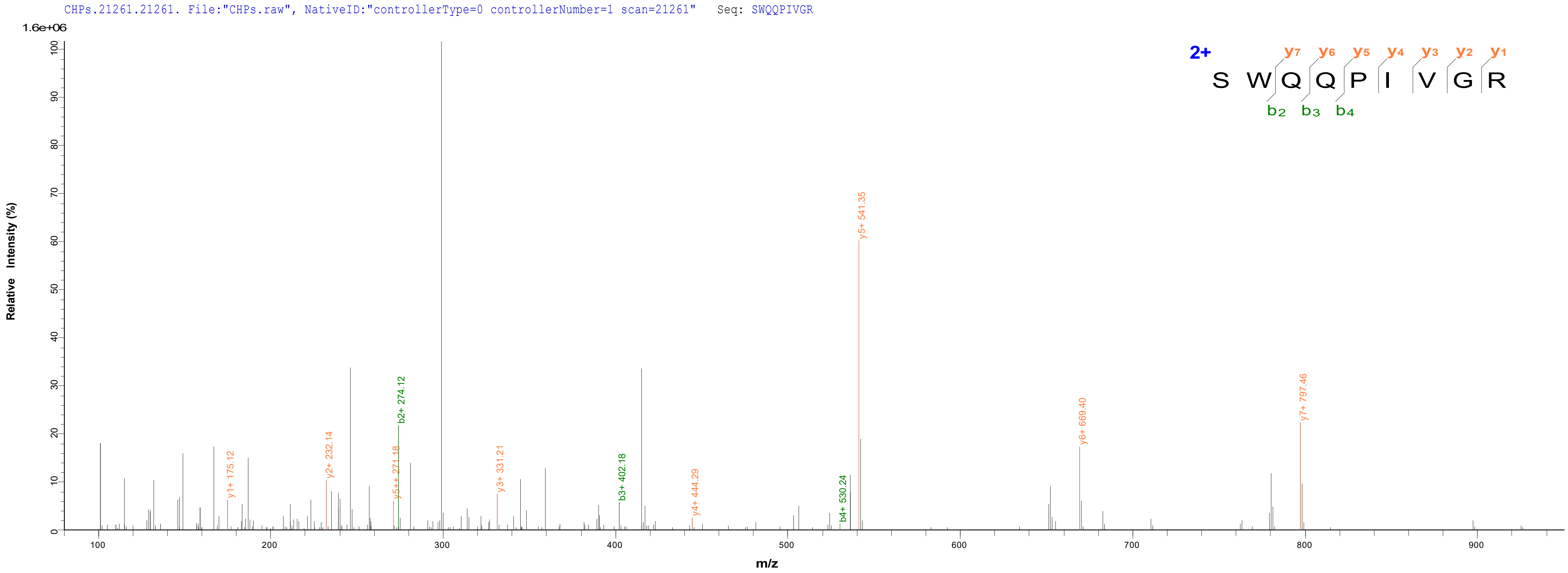

CHPs.21261.21261. File:"CHPs.raw", NativeID:"controllerType=0 controllerNumber=1 scan=21261" Seq: SWQQPIVGR
1.6e+06
Relative Intensity (%)
m/z
2+
S W Q Q P I V G R
y7 y6 y5 y4 y3 y2 y1
b2 b3 b4
y1+ 175.12
y2+ 232.14
y5++ 271.18
b2+ 274.12
y3+ 331.21
b3+ 402.18
y4+ 444.29
b4+ 530.24
y5+ 541.35
y6+ 669.40
y7+ 797.46
